# Extreme infectious titer variability in individual *Aedes aegypti* mosquitoes infected with Sindbis virus is associated with both differences in virus population structure and dramatic disparities in specific infectivity

**DOI:** 10.1371/journal.ppat.1012047

**Published:** 2024-02-27

**Authors:** Peter Hodoameda, Gregory D. Ebel, Suchetana Mukhopadhyay, Rollie J. Clem

**Affiliations:** 1 Division of Biology, Kansas State University, Manhattan, Kansas United States of America; 2 Department of Microbiology, Immunology and Pathology, Colorado State University, Fort Collins, Colorado United States of America; 3 Department of Biology, Indiana University, Bloomington, Indiana United States of America; Washington State University, UNITED STATES

## Abstract

Variability in how individuals respond to pathogens is a hallmark of infectious disease, yet the basis for individual variation in host response is often poorly understood. The titer of infectious virus among individual mosquitoes infected with arboviruses is frequently observed to vary by several orders of magnitude in a single experiment, even when the mosquitoes are highly inbred. To better understand the basis for this titer variation, we sequenced populations of Sindbis virus (SINV) obtained from individual infected *Aedes aegypti* mosquitoes that, despite being from a highly inbred laboratory colony, differed in their titers of infectious virus by approximately 10,000-fold. We observed genetic differences between these virus populations that indicated the virus present in the midguts of low titer mosquitoes was less fit than that of high titer mosquitoes, possibly due to founder effects that occurred during midgut infection. Furthermore, we found dramatic differences in the specific infectivity or SI (the ratio of infectious units/viral genome equivalents) between these virus populations, with the SI of low titer mosquitoes being up to 10,000-fold lower than that of high titer mosquitoes. Despite having similar amounts of viral genomes, low titer mosquitoes appeared to contain less viral particles, suggesting that viral genomes were packaged into virions less efficiently than in high titer mosquitoes. Finally, antibiotic treatment, which has been shown to suppress mosquito antiviral immunity, caused an increase in SI. Our results indicate that the extreme variation that is observed in SINV infectious titer between individual *Ae*. *aegypti* mosquitoes is due to both genetic differences between virus populations and to differences in the proportion of genomes that are packaged into infectious particles.

## Author summary

Individual hosts typically respond differently to virus infection. These differences are usually attributed to genetic variation between hosts or differences between virus strains. However, when highly inbred mosquitoes are infected with an arbovirus, individual mosquitoes still often vary widely in titer of infectious virus. We examined the virus populations in individual *Aedes aegypti* mosquitoes infected with Sindbis virus that differed by approximately 10,000-fold in the amount of infectious virus. Based on our experiments, we have made two important findings. First, we found genetic differences between these virus populations, suggesting that viruses in low titer mosquitoes were less fit than those in high titer mosquitoes, potentially due to genetic founder effects. Second, we also found that the amounts of viral genomes did not vary significantly between low and high titer mosquitoes, despite huge differences in the amount of infectious virus. Furthermore, the proportion of viral genomes packaged into infectious particles was improved by feeding the mosquitoes antibiotics, which has been shown to suppress antiviral immunity. These findings have important implications regarding our understanding of the transmission of arboviral disease.

## Introduction

Mosquito-transmitted arboviruses continue to take a substantial negative toll on public health worldwide, making them a significant global public health threat [1]. A major factor contributing to the public health threat associated with arboviruses is due to their ability to emerge in new geographical locations or re-emerge at previously existing locations. Factors such as changes in mosquito anthropological behaviors, changing climatic conditions and the appearance of arbovirus genetic variants are some of the important contributing factors to arbovirus emergence and re-emergence [2].

The low fidelity of viral RNA-dependent RNA polymerases introduces frequent mutations into the genomes of RNA viruses during replication [3] and alphaviruses such as Sindbis virus (SINV) are no exception [4,5]. The mutant swarms that result due to errors in viral replication produce many variants with low fitness, but at the same time these diverse genetic populations are important in making the virus more adaptable and able to replicate more efficiently in both the mosquito vector and the vertebrate host [3]. It is thought that the requirement for arboviruses to replicate in the diverse environments of the mosquito vector and vertebrate host, coupled with the multiple severe genetic bottlenecks that arboviruses experience in both vector and host, are major factors in maintaining their genetic stability over time [6–8].

For arboviruses to successfully establish infection in the mosquito, they need to traverse anatomical barriers including the midgut and salivary glands [9]. These anatomical barriers, such as those associated with infecting and escaping the midgut and the salivary glands, exert strong genetic bottleneck effects on arboviruses [10–13]. Thus, during a single round of infection in a mosquito, individual sequence variants inevitably increase or decrease in frequency in a virus population due to these bottlenecks, which can be severe. For example, using low doses of marked clones of Venezuelan equine encephalitis virus (VEEV), it was shown that infection of *Culex taeniopus* results in two severe bottlenecks, each narrowing the virus population down to less than 10 virus particles that initiate midgut infection and escape the midgut, respectively [11]. The severe reduction in genetic diversity caused by these bottlenecks can cause stochastic founder effects, where the resulting population after a bottleneck differs genetically from the original population. However, genetic diversity is rapidly restored during subsequent rounds of virus replication because of high error rates [13].

The titer of infectious virus in a mosquito is potentially of epidemiological importance because there is likely a positive correlation between virus titer in the mosquito and transmission risk. For example, in a study examining infection of *Aedes aegypti* by another alphavirus, chikungunya virus, it was found that for each 10-fold increase in viral titer in the mosquito legs there was a 49% increase in the probability of detecting virus in the saliva [14]. Interestingly, numerous studies have documented that there is often extensive variation in arbovirus titer between individual mosquitoes after being given an infectious blood meal. This huge titer variation, which is often up to several orders of magnitude, is commonly observed in both field-caught mosquitoes and mosquitoes from highly inbred laboratory colonies [15–18]. While this variation may not be so surprising in field-caught mosquitoes, which are relatively genetically diverse, the potential explanation for this extreme titer variation is less obvious when highly inbred mosquitoes are infected. Despite the importance of mosquito titer in vector competence, until recently, there has been little attention paid to individual titer variation in arbovirus-infected mosquitoes [16]. One study published in 2017 examined transcriptomes in individual *Ae*. *aegypti* during infection with dengue virus (DENV) and identified several candidate genes whose expression levels correlated with virus RNA load, some of which affected DENV RNA levels when their expression was knocked down [18]. However, because the study was done using mosquitoes that had been recently field-caught, it was presumed by the authors that genetic differences between individuals was the primary cause of the variation in DENV RNA load that was observed [18].

In a previous study, we began to investigate the causes of the extreme titer variation that exists between individual mosquitoes following SINV infection of highly inbred laboratory strains of *Ae*. *aegypti* [16]. Our work focused mainly on the Orlando strain, which has been in continuous culture since around 1940, with limited outcrossings up until 1992 [19]. Our Orlando colony has not been outcrossed since at least 2003. Despite this high degree of inbreeding, we routinely observe titer differences of several logs in a single experiment when using Orlando mosquitoes. This large amount of titer variation was not affected by individual mosquito size, the amount of blood ingested, the concentration of infectious virus fed to the mosquitoes, or the age of the mosquitoes when they were infected [16]. The only two factors that were identified as affecting titer variability were the route of infection, with less variation being observed following intrathoracic versus oral infection, and the incubation period after infection, with overall virus titer decreasing and becoming less variable over time [16]. The observation that virus titers are less variable when the midgut is bypassed by intrathoracic injection suggests that the midgut infection and escape barriers and their associated genetic bottlenecks may play a role in causing individual variation. Interestingly, in the same study, we showed that SINV obtained from high titer mosquitoes replicated to higher titers in cultured cells compared to virus from low titer mosquitoes. This result further suggested that there may be genetic differences between the virus populations in low and high titer mosquitoes contributing to these phenotypic differences.

In this study, we sequenced SINV populations from individual high and low titer *Ae*. *aegypti* mosquitoes that had been infected as a single cohort, in order to investigate whether viral genetic factors contribute to virus titer variation after oral infection. Significant differences were observed between the virus populations obtained from low titer mosquitoes versus those from high titer mosquitoes, including stronger reductions in genetic complexity and diversity during dissemination from the midgut and evidence of stronger negative selection occurring in low titer mosquitoes. In addition, we also found that the specific infectivity (SI, or the ratio of infectious virus to viral genome equivalents) of these virus populations was up to 10,000-fold higher in high titer mosquitoes than in low titer mosquitoes, indicating that titer variability in this arbovirus-vector combination is primarily due to dramatic differences in SI.

## Results

### SINV titer is highly variable and correlates with detection of virus in saliva

**Fig 1:**
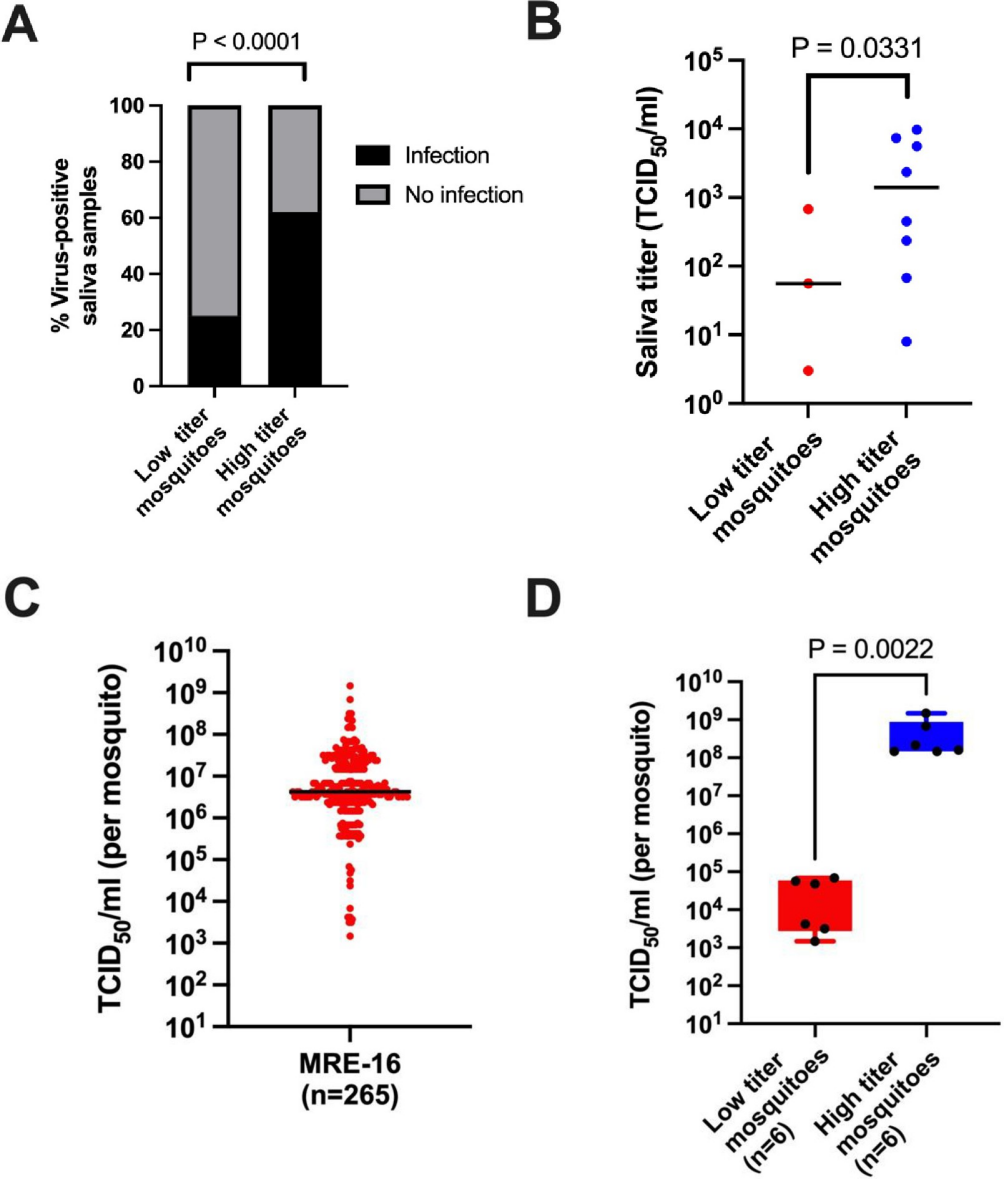
Oral infection of *Ae*. *aegypti* with SINV results in highly variable titers, and virus titer correlates with the presence of infectious virus in saliva. (A) The proportions of virus-positive saliva samples collected from low titer and high titer mosquitoes. (B) Infectious titers of the virus-positive saliva samples in panel A. (C) Titers of mosquitoes collected at 5 days PBM (n = 265). (D) The titers of the 12 low and high titer mosquitoes selected for sequencing. Fisher’s exact test was used in panel A, while Mann-Whitney test was used in panels B and D.

We and others have repeatedly shown that extreme variation in virus titer is observed between individual mosquitoes that are infected with various arboviruses [15–17,20,21], even when the mosquitoes are highly inbred. We first asked whether titer variation affects the likelihood of virus being detected in the saliva of infected mosquitoes, which is often used as a proxy to assess transmission potential. Saliva was collected at 5 days post-blood meal (PBM) from *Ae*. *aegypti* (Orlando) mosquitoes that had fed on blood containing passage 2 (P2) virus derived from p5’dsMRE16ic, an infectious clone of the MRE-16 strain of SINV. After determining the virus titers in the mosquito bodies by tissue culture median infectious dose (TCID_50_) assay, the infectious titers in the saliva samples from 13 high titer (≥10^8^ TCID_50_/mL) and 12 low titer (≤10^4^ TCID_50_/mL) mosquitoes were determined. A significantly larger proportion of high titer mosquitoes had detectable virus in their saliva than low titer mosquitoes (Fig 1A). Furthermore, amongst those mosquitoes that had positive saliva titers, the median saliva titer was larger in high versus low titer mosquitoes (Fig 1B). Based on these results, it is likely that individual variation in carcass titer can affect virus transmission.

We previously found that SINV collected from high titer mosquitoes replicated to a higher titer in cultured cells than virus from low titer mosquitoes [16], suggesting there may be genetic differences between these virus populations. To further examine this, we performed Illumina sequencing on virus populations from high and low titer mosquitoes. Mosquitoes were allowed to ingest blood containing 1.6×10^9^ TCID_50_/mL of P2 virus derived from p5’dsMRE16ic. At 5 days PBM, we dissected the mosquitoes into midguts and carcasses (defined as the rest of the mosquito besides the midgut) and determined the virus titers of the dissected mosquito carcasses using TCID_50_ assay. Consistent with previous results, the individual carcass titers varied widely; the median carcass titer was 4.2x10^6^ TCID_50_/mL, but individual titers ranged from 1.5x10^9^ to 3.7x10^3^ (Fig 1C). From this group of mosquitoes, we selected six high titer (≥1.5x10^8^ TCID_50_/mL) and six low titer (≤6.8x10^4^ TCID_50_/mL) mosquitoes for Illumina sequencing of their individual midguts and carcasses, in order to compare the sequences of these individual virus populations (Fig 1D).

### A significant reduction in numbers of sequence variants occurs following dissemination from midgut to carcass, but only in low titer mosquitoes

**Fig 2:**
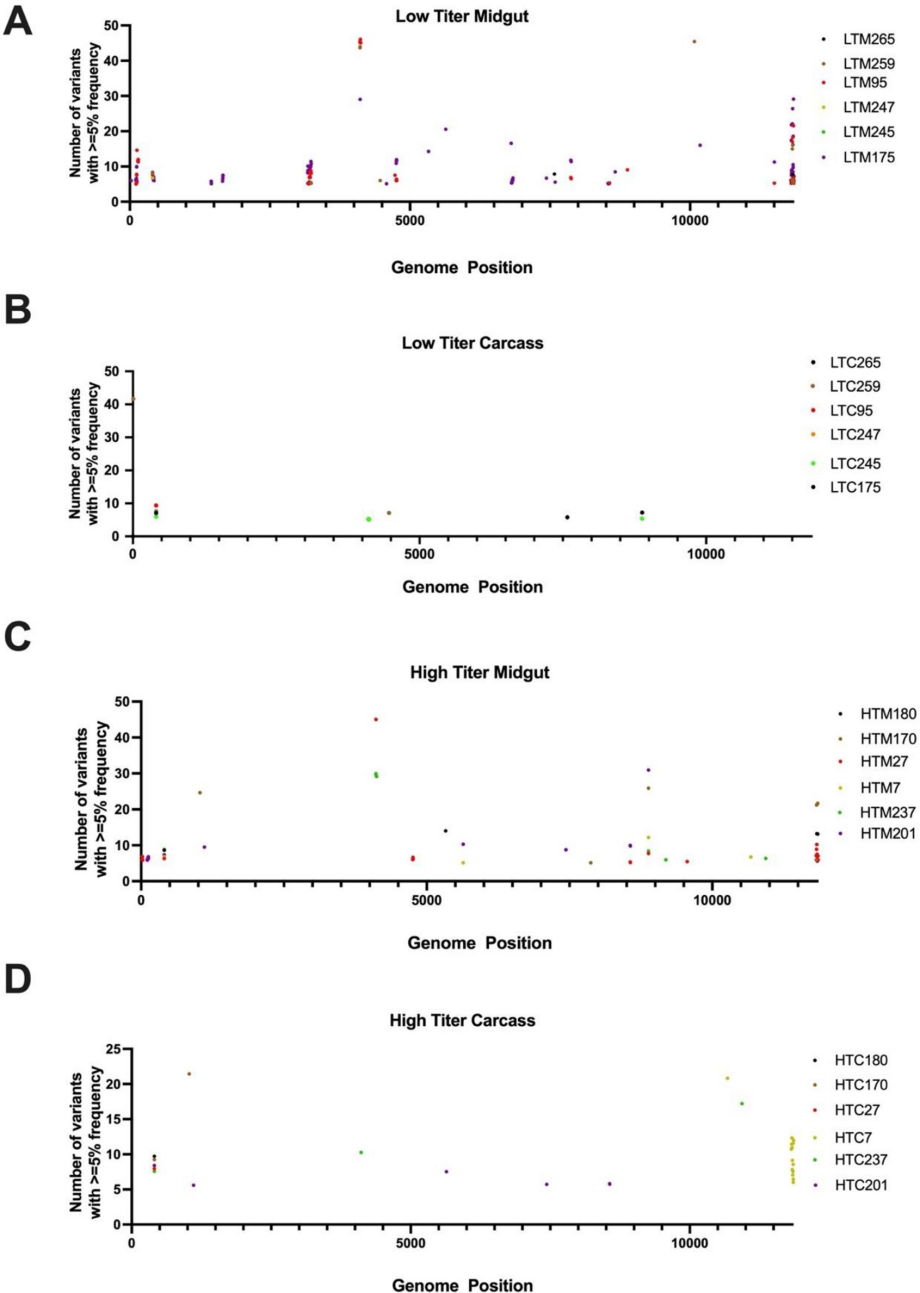
SINV variants with frequency ≥5% in (A) midgut and (B) carcass of low titer mosquitoes, and (C) midgut and (D) carcass of high titer mosquitoes. The individual mosquito sample identifiers are listed to the right of each panel. LTM, low titer midgut; LTC, low titer carcass; HTM, high titer midgut; HTC, high titer carcass.

RNA isolated from the individual midgut and carcass samples of the 6 low and 6 high titer mosquitoes, as well as from a sample of the P2 virus stock that was fed to the mosquitoes, was used to prepare Illumina sequencing libraries, which were then subjected to sequencing. The resulting viral sequences were aligned and compared to the reference sequence of p5’dsMRE16ic. Viral genome sequence coverage in the mosquito tissue samples ranged from approximately 2,000X to 18,000X, with the exception of one low titer carcass sample which was around 40X, while genome coverage in the stock sample was around 25,0000X (S1 Fig). To reveal significant sequence changes, we focused on nucleotide variants that were ≥5% in frequency within a sample. Overall, the amount of genetic diversity observed between samples was relatively low (positions of variants present at ≥5% are listed in S1 Data). Very few indels were observed, with a total of only 17 indels being ≥5% in frequency in all samples combined, and those observed were small (≤3 nucleotides) (S2 Fig). Nucleotide substitutions were distributed in what appeared to be a random pattern across the genome, although there were a few apparent “hot spots”, or sites that contained higher numbers of variants (Fig 2; sequence variants in stock virus are shown in S3 Fig).

**Fig 3:**
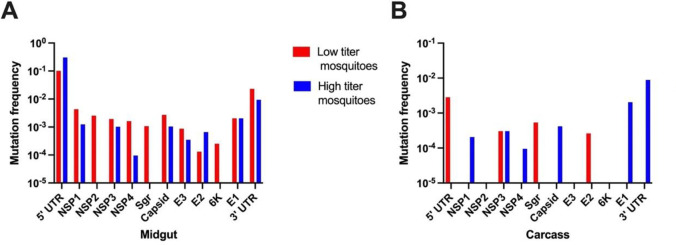
Distributions of nucleotide variants with frequency ≥5% in the genomes of virus derived from (A) midguts and (B) carcasses. Mutation frequency was calculated by dividing the number of variants within a given region by the length of the region. Non-structural proteins are represented by NSP1 to NSP4 while the structural proteins are represented by capsid, E3, E2, E1, and 6K. Variants present in the 5’ UTR, sub-genomic promoter (sgr), and 3’ UTR regulatory elements are also shown.

To examine whether there were differences in the degree of sequence variation amongst different viral ORFs, we first calculated the mutation frequencies (average mutations per nucleotide position) in order to account for differences in ORF length. The genome positions of ORFs and other genomic elements are provided in S1 Table. Mutation frequencies were roughly similar in most of the 9 viral ORFs in midguts of low and high titer mosquitoes (Fig 3A), except that no mutations were identified at ≥5% frequency in the NSP2 and 6K ORFs of high titer midguts. Greater variability was seen in carcasses, where three ORFs (NSP2, E3, and 6K) had no mutations at ≥5%, and mutations at ≥5% were only seen in NSP3 and E2 of low titer carcasses. Mutation frequency varied somewhat in the 5’ or 3’ untranslated regions and the subgenomic promoter region, but the relatively small sizes of these regions and overall low number of mutations impacts the interpretation of these results.

Importantly, no single mutations were observed that correlated with virus titer. Mutations observed in the different samples were to a large extent unique, with a minority being shared between samples (S4 Fig). There were also no clear patterns of differences in the proportions of mutations that were synonymous versus non-synonymous in the ORFs of viruses from low versus high titer mosquitoes (S5 Fig).

**Fig 4:**
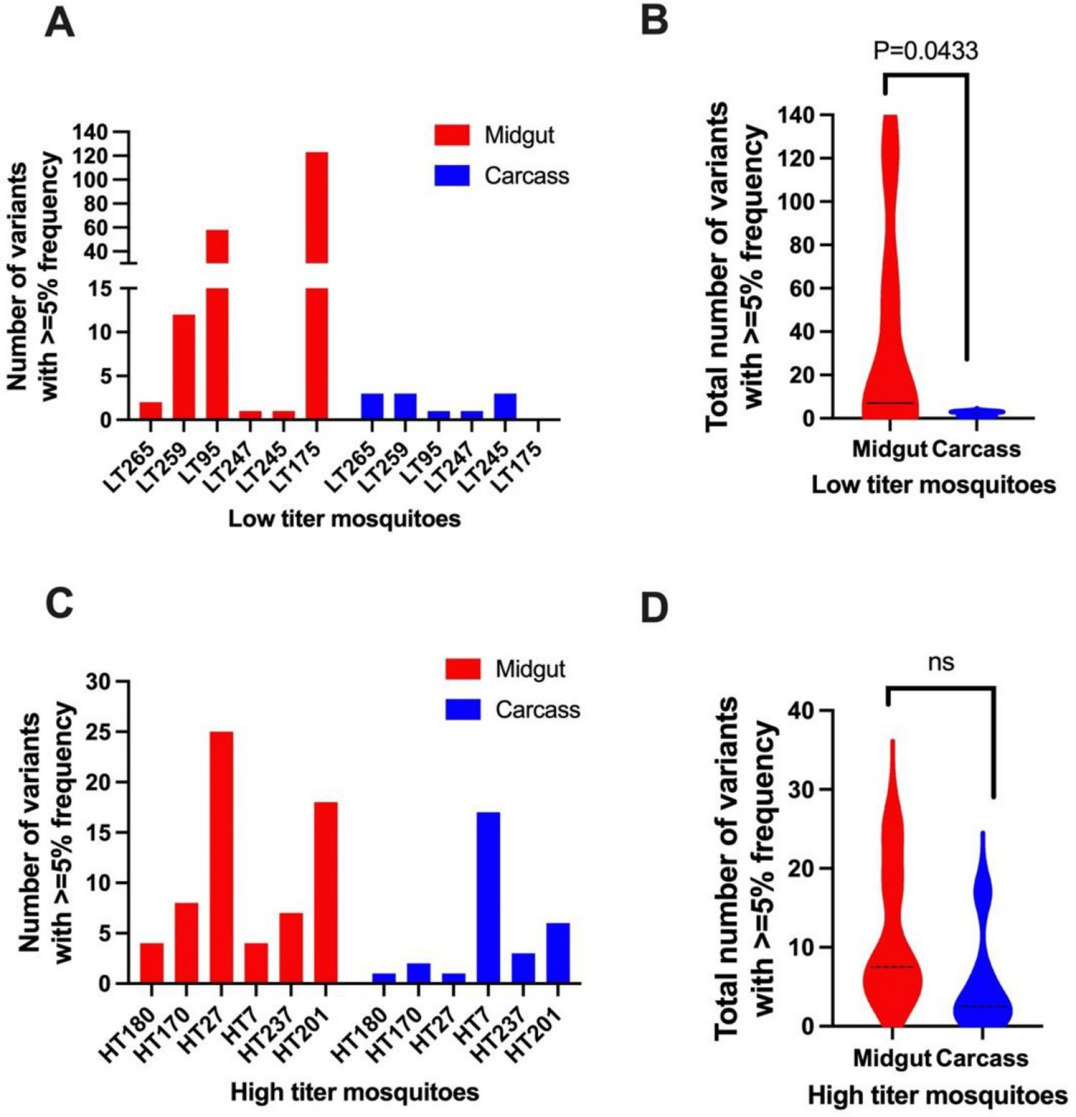
Reductions in the numbers of variants following midgut escape in low and high titer mosquitoes. The numbers of sequence variants in midguts and carcasses of individual low titer (A) and high titer (C) mosquitoes are shown, along with the total number of variants in midguts and carcasses of low titer (B) and high titer (D) mosquitoes. Mann-Whitney test was used for statistical analysis in panels B and D.

Genetic bottlenecks exist for arboviruses at the levels of midgut infection and midgut escape. Consistent with a strong bottleneck occurring during midgut escape, we observed that the numbers of sequence variants were reduced in the carcasses compared to the midguts of three of the six low titer mosquitoes, resulting in an overall significant reduction in the number of variants when low titer individuals were taken as a whole (Fig 4A and 4B). Interestingly, however, there was no significant difference in the number of sequence variants between the midguts and carcasses of the high titer mosquitoes (Fig 4C and 4D). This result suggests that there was more of a reduction in genetic complexity, or in other words a more stringent bottleneck, associated with virus dissemination in low titer mosquitoes than in high titer mosquitoes. However, there was no significant difference between low titer and high titer mosquitoes in terms of the numbers of variants that were observed in both midgut and carcass (conserved variants) or the numbers of variants seen only in carcass samples (emerged variants) (S6 Fig). There was also no significant difference observed in bottleneck size when neutral markers were used to estimate the sizes of the bottlenecks associated with dissemination from midgut to carcass in low and high titer mosquitoes (S7 Fig). Thus, although there was evidence of a more stringent genetic bottleneck in low titer than in high titer mosquitoes, bottleneck size did not appear to differ between these groups.

### Differences in diversity and complexity between the virus populations in low titer and high titer mosquitoes

**Fig 5:**
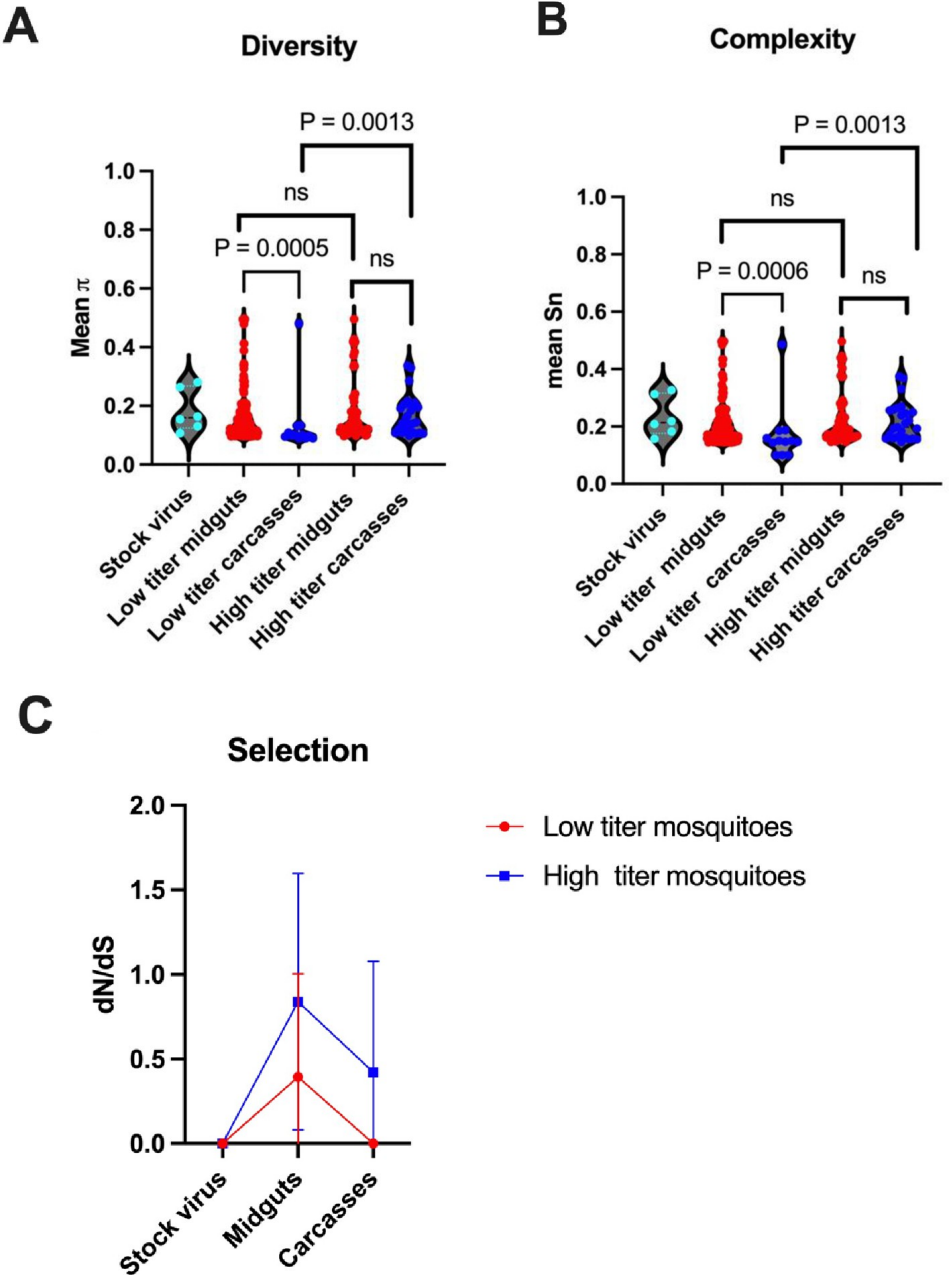
Genetic diversity, complexity, and purifying selection in low and high titer mosquitoes. Both (A) diversity and (B) complexity was reduced in virus populations found in carcasses compared to midguts of low titer mosquitoes, but not of high titer mosquitoes. (C) Stronger purifying selection was observed in both midguts and carcasses of low titer mosquitoes than in high titer mosquitoes. Mann-Whitney test was used for statistical analysis in panels A and B. SINV variants with frequency ≥5% were used in the analysis.

In order to gain a better understanding of the overall genetic differences between the various virus populations, we then compared the diversity (a measure of the degree of sequence variation) and complexity (a broader measure of variability, including richness, diversity and heterogeneity) of the virus populations in the cumulative midguts and carcasses of low and high titer mosquitoes, as well as to those of the original stock virus that was used to infect the mosquitoes. This analysis showed that there was a significant reduction in both sequence diversity and complexity after dissemination from midgut to carcass in low titer mosquitoes, but not in high titer mosquitoes (Fig 5A and 5B). In addition, we examined the ratio of non-synonymous to synonymous substitutions (the *d*_*N*_/*d*_*S*_ ratio) in the coding regions of the virus populations, in order to assess the strength of natural selection in these virus populations. A *d*_*N*_/*d*_*S*_ ratio of less than 1 indicates that negative (purifying) selection is occurring in the population. This analysis revealed that purifying selection occurred in all the mosquito-derived virus populations, but the magnitude of the purifying selection was greater in both midguts and carcasses of low titer mosquitoes when compared to high titer mosquitoes (Fig 5C). Together, these analyses indicate that there was a stronger reduction in virus genetic diversity and complexity, accompanied by stronger purifying selection, during the process of virus dissemination from midgut to carcass in low titer versus high titer mosquitoes.

### Low titer mosquitoes contain less viral particles and lower levels of capsid protein than high titer mosquitoes

Although we observed differences at the population genetic level between the virus populations in low and high titer mosquitoes, it seemed unlikely that these differences alone could cause the ~10,000-fold difference observed in infectious virus titers between low and high titer mosquitoes. To first rule out the possibility that titer was affected by the type of cells used to assay infectious virus, we compared TCID_50_ values using mammalian BHK and mosquito Aag2 and C6/36 cells. Mosquito samples that had high or low titers, as measured in BHK cells, were re-titered in mosquito cells using an immunofluorescence-based TCID_50_ assay. TCID_50_ values were highly consistent regardless of the cell line used (S8 Fig), indicating that the type of cells used for virus titration does not affect the measurement of infectious virus.

We reasoned that the large differences in virus titer between individuals could be due to various reasons, including differences in the amounts of viral RNA (due to differences in synthesis or stability), differences in the amounts of viral proteins being translated from equivalent levels of viral RNA, differences in the proportion of viral RNA being packaged into particles, or the presence of large numbers of defective virus particles. To begin to decipher the basis for these large differences in titer, we orally infected another batch of mosquitoes and, after homogenization in PBS and titration by TCID_50_, randomly selected three high titer, three medium titer, and three low titer mosquitoes in order to look for the presence of virus particles by transmission electron microscopy (TEM). A portion of the clarified lysate from the high, medium and low titer samples was negative stained and examined by TEM. Enveloped icosahedral virus particles consistent with the known size of SINV (~70 nm) were abundantly observed in homogenates from all three of the high titer mosquitoes we examined (S9 Fig and S2 Table). However, we rarely observed virus particles during examination of homogenates from 3 medium titer and 3 low titer mosquitoes (S2 Table). Obviously, there must have been virus particles present in the low and medium titer samples, but they were apparently too dilute to be consistently observable by this technique, consistent with their lower TCID_50_ values. Thus, based on this semi-quantitative method, it appeared that there were less viral particles in low and medium titer mosquitoes than in high titer mosquitoes. It should also be noted that that we did not observe any unenveloped nucleocapsid cores by TEM in any of the samples.

We next used immunoblotting to examine the levels of viral capsid protein in low and high titer mosquitoes. An additional batch of mosquitoes were orally infected and homogenized in PBS, and after a small portion of each homogenized sample was analyzed by TCID_50_ assay, 7 high titer and 7 low titer mosquitoes were chosen for immunoblotting using antibodies against SINV capsid protein and glycoproteins E1 and E2. The results showed that, while there was variability in the levels of capsid protein between individuals, on average there was 1.6-fold less capsid protein in low titer compared to high titer mosquitoes (S10 Fig). Although we were unable to detect E1 and E2 in this assay, this may have simply been a detection issue given the small amount of starting material in single mosquitoes, since in our experience the glycoproteins are more difficult to detect than capsid. Nevertheless, although levels of capsid protein would not necessarily need to be reduced in order to result in lower numbers of assembled virions, the reduced capsid protein levels in low titer mosquitoes is consistent with the lower numbers of virions observed by TEM.

### Dramatic differences in specific infectivity (SI) between high titer and low titer mosquitoes

**Fig 6:**
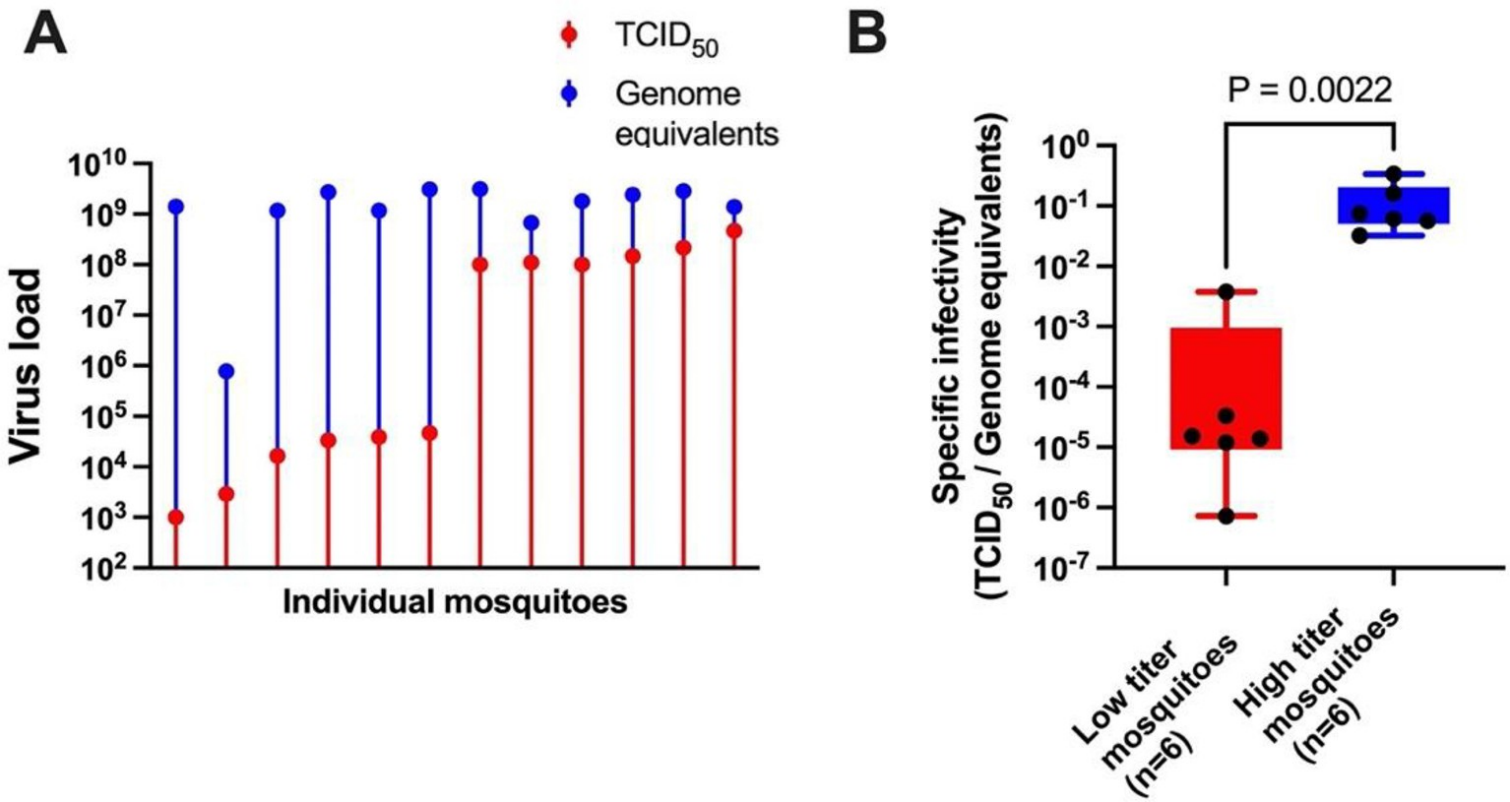
The specific infectivity (SI) ratios are drastically different between the carcasses of the 6 low titer versus the 6 high titer mosquitoes used for sequencing. (A) Genome equivalents and virus titers in carcasses of the individual sequenced low titer and high titer mosquitoes. (B) Specific infectivity in carcasses of the individual low titer and high titer mosquitoes. Mann-Whitney test was used to analyze the results in (B).

Since there appeared to be lower numbers of viral particles in low titer mosquitoes, it was important to determine the levels of viral RNA in the two groups, in order to distinguish between differences in viral genome replication versus differences in the proportion of genomes packaged into infectious particles. To examine this, we determined the SI (the ratio of infectious units to viral genomes) of virus populations from individual mosquitoes, initially from the same 6 low and 6 high titer mosquitoes that were used for sequencing. SINV genome equivalents were measured by RT-qPCR using primers that amplified a portion of the NSP3 region, and were compared to infectious units as measured by TCID_50_ assay in the same mosquitoes. Interestingly, we found that genome equivalents did not vary to any great extent in 11 of the 12 mosquitoes, even though the infectious units were considerably different between the low and high titer mosquitoes (Fig 6A). This resulted in a large difference in SI (TCID_50_/genome equivalents) between the two groups of mosquitoes (Fig 6B).

**Fig 7:**
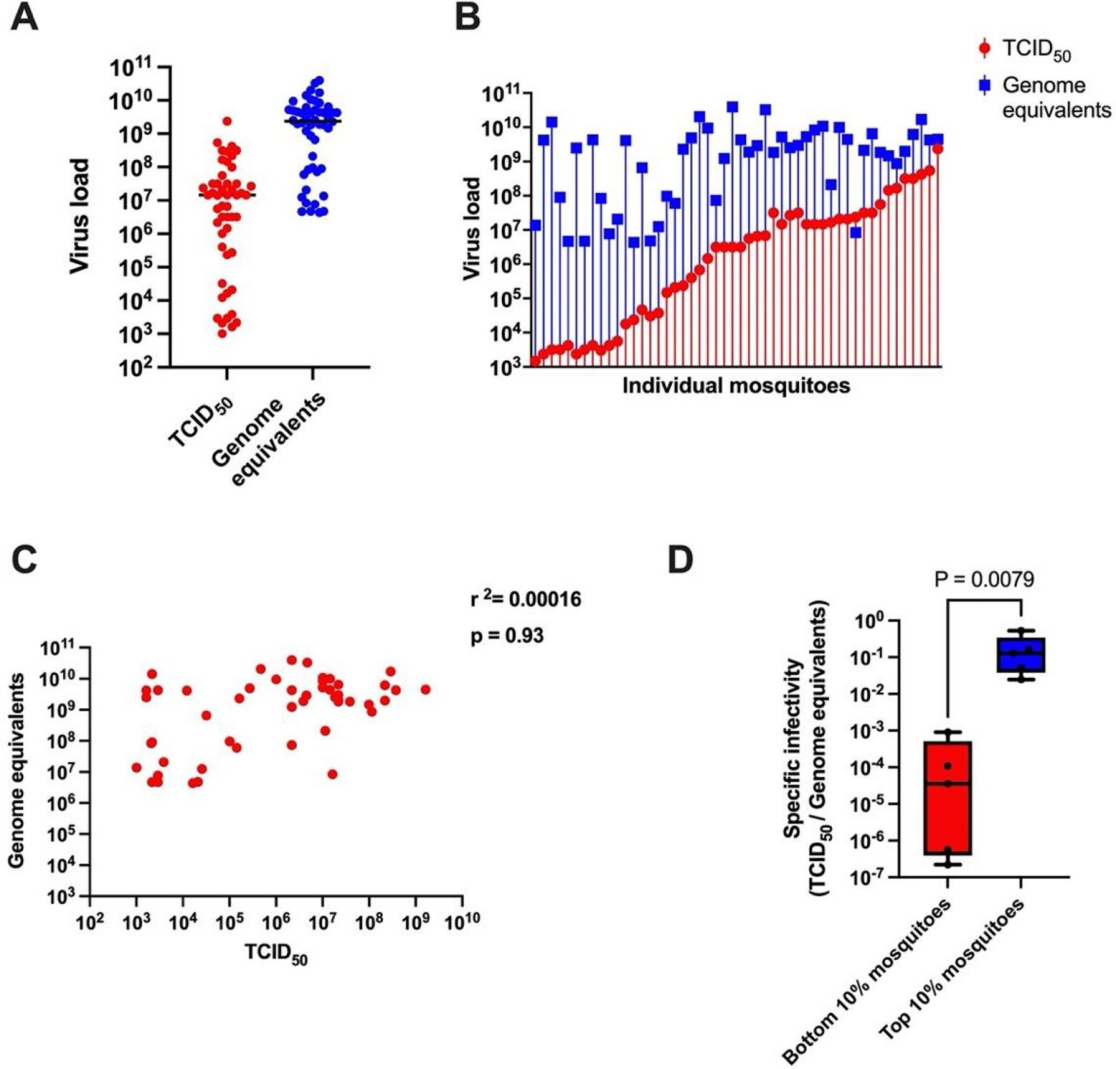
Dramatic differences are observed in SI between individual mosquitoes (n = 50). (A) Genome equivalents and virus titers in individual mosquitoes. (B) Genome equivalents compared to their corresponding virus titers, arranged in ascending order of titer. (C) No significant correlation was observed between genome equivalents and virus titers. (D) SI values of the mosquitoes with the lowest 10% and highest 10% of titers. Pearson correlation test was used in panel C, while Mann Whitney test was used in D.

To determine whether this dramatic difference in SI was a general phenomenon and not restricted to this small group of mosquitoes, another batch of 50 mosquitoes were infected by blood feeding as before, and the SI of these individual mosquitoes was determined. Consistent with what was observed in the original 12 sequenced mosquitoes, we observed less variation in genome equivalents compared to infectious units amongst these mosquitoes (Fig 7A and 7B). Overall, the numbers of genome equivalents were greater than infectious units in almost all individuals (Fig 7B). Although this would be expected, in part since RT-PCR is more sensitive than TCID_50_ assay, and also because all viruses produce more genomes than infectious particles, we noted that the difference between genome equivalents and virus titer in low titer individuals was strikingly large (Fig 7B). Genome equivalents also varied to some degree, but there was no significant overall correlation between genome equivalents and TCID_50_ values (Fig 7C), with many individuals that had low titers of infectious virus containing high numbers of genomes. Notably, the top 10% of mosquitoes in terms of infectious titers had SI values ranging from 0.03 to 0.53, while the bottom 10% ranged from 5.6x10^-7^ to 9.1x10^-4^ (Fig 7D). These results, together with our TEM and immunoblot data, indicate that the large variation that is observed in individual mosquito titer is mainly driven by differences in the proportion of genomes that are packaged into infectious virus particles, rather than by differences in the levels of viral genomic RNA.

### Carcass SI is significantly higher than midgut SI

**Fig 8:**
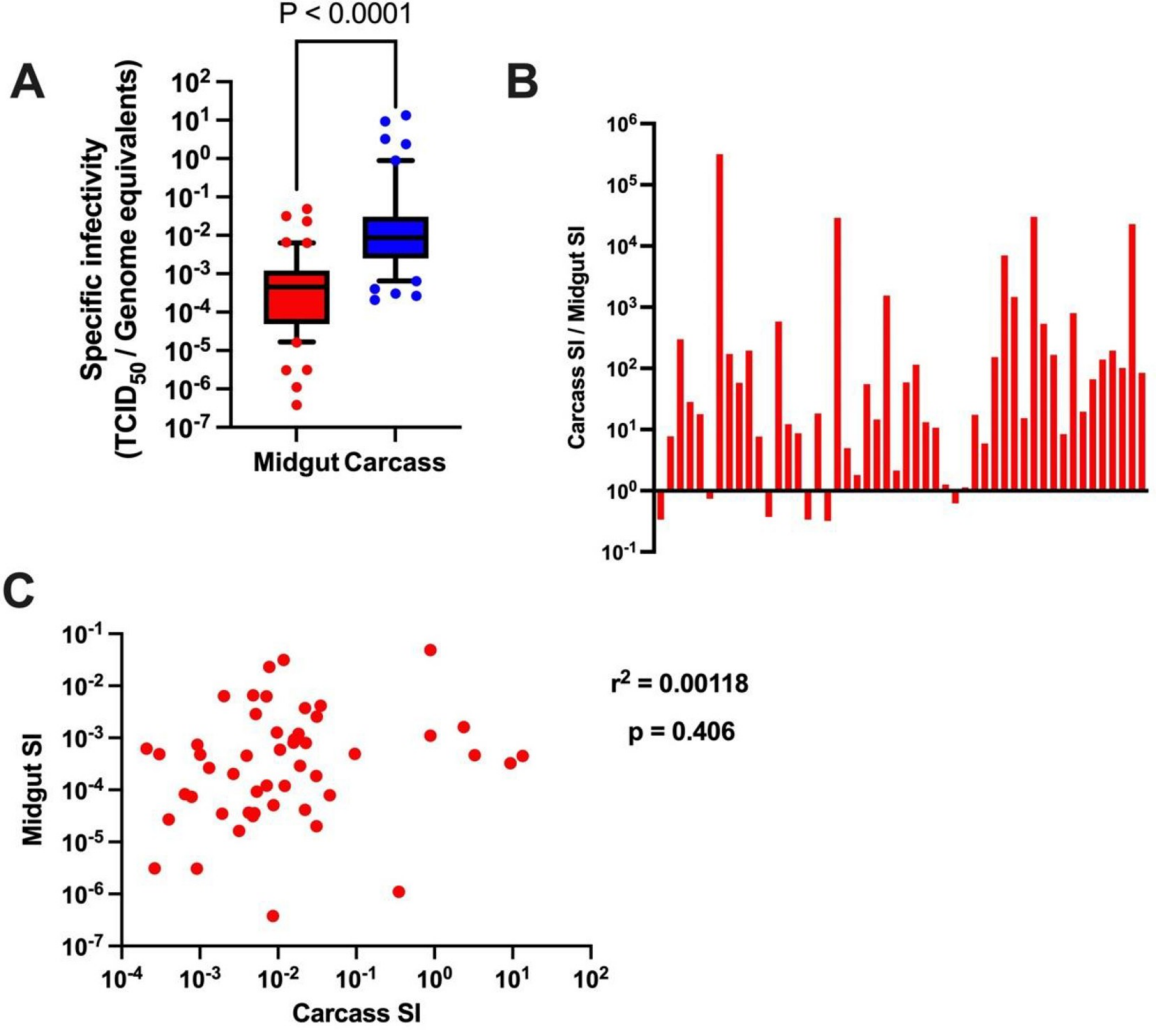
Midgut SI is lower than carcass SI in individual mosquitoes (n = 50). (A) Box and whisker plot of midgut and carcass specific infectivity. (B) Ratio of carcass SI to midgut SI in individual mosquitoes, showing that carcass SI is higher than midgut SI in the majority of mosquitoes. (C) Pearson correlation analysis revealed a lack of significant correlation between midgut SI and carcass SI. Mann-Whitney test was used in panel A.

Our sequence analysis indicated that there were significantly greater reductions in genetic diversity and complexity occurring during virus dissemination in low versus high titer mosquitoes. It was therefore of interest to compare the SI values in carcasses and midguts. To do this, another batch of 50 mosquitoes were orally infected as before. All mosquitoes had detectable virus titer in both midgut and carcass, indicating that variation in midgut titer does not affect the dissemination rate. When we compared midgut and carcass SI values in the individual mosquitoes, we again found that viral RNA levels did not correspond to infectious titer in either tissue (S11 Fig). In addition, overall carcass SI was significantly higher than midgut SI (Fig 8A). Carcass SI values were greater than midgut SI in the large majority (88%) of individual mosquitoes (Fig 8B). Thus, there tended to be a higher ratio of non-infectious to infectious genomes in the midgut than in the carcass of most individuals. However, there was no significant correlation between midgut SI and carcass SI in individuals (Fig 8C), indicating that the magnitude of midgut SI does not appear to influence the SI of disseminated virus within an individual infected mosquito.

### SI decreases with increased incubation period

Previously published results from our group showed that SINV titer variation decreases with time after an infectious blood meal (the incubation period); there was significantly decreased median titer as well as less titer variation at 15 days PBM than at earlier times [16]. To ask whether this reduction in titer variation with increased incubation period is also associated with differences in SI, we compared the SI of mosquitoes at 5 days and 15 days PBM. The results indicated that there was significantly lower SI at 15 days PBM than at 5 days PBM (S12 Fig). Thus, the overall reduction in virus titer over time that was previously observed also appears to be due to accumulation of unpackaged genomes.

### Oral antibiotic treatment results in higher specific infectivity

**Fig 9:**
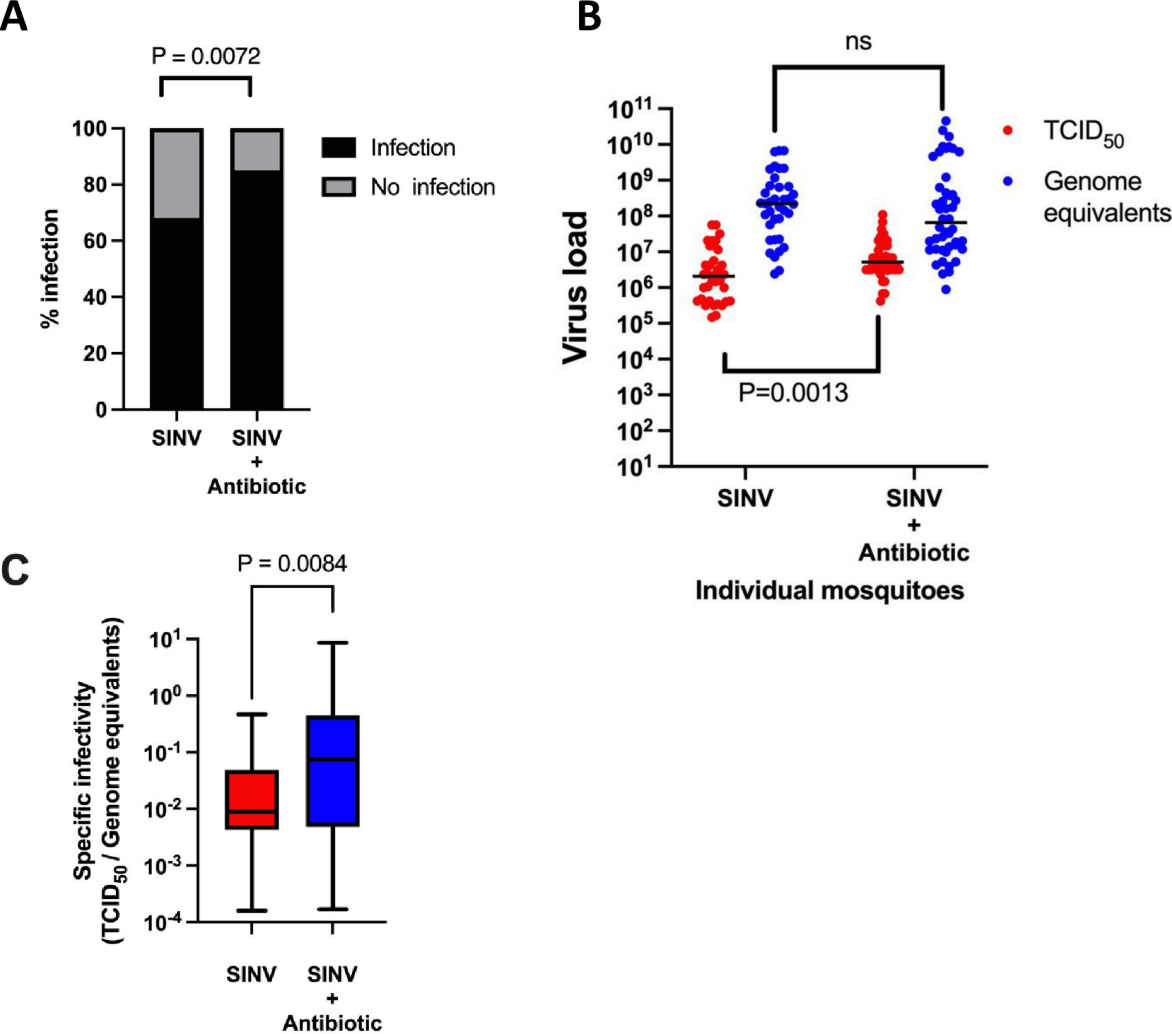
Oral treatment of mosquitoes with antibiotics results in higher titers of infectious virus, but does not affect viral genome levels. (A) The proportion of mosquitoes with detectable infectious virus is increased by antibiotic treatment (n = 54 for antibiotic-treated and 56 for control). (B) Despite causing an increase in infectious titer, antibiotic treatment does not affect viral genome levels. (C) Specific infectivity is increased by antibiotic treatment. Fisher’s exact test was used in panel A, Mann-Whitney test was used in panels B and C.

It has been shown previously that feeding antibiotics to mosquitoes suppresses antiviral immunity due to the disruptive effects of antibiotics on the composition of the gut microbiome [22–24]. To test whether genome packaging efficiency is influenced by altering the immune response, we exposed pupae and adults to antibiotics in their water source prior to also providing antibiotics in an infectious blood meal. As expected based on previous reports, antibiotic treatment resulted in increases in both infection prevalence and median infectious titer compared to mosquitoes that were not exposed to antibiotics, likely due to the previously demonstrated immune suppressive effects of antibiotics (Fig 9A and 9B). However, antibiotic treatment did not significantly affect viral genome levels (Fig 9B). The resulting increase in median SI (Fig 9C) indicates that suppressing the immune response results in a higher proportion of viral genomes being packaged into infectious particles.

## Discussion

Variation in how individual hosts respond to infections has been known to exist since antiquity; for example, some hosts survive while others succumb to infection, and symptom severity can vary widely between individuals. However, most of the past research on this topic has focused on genetic differences between hosts or differences in virulence genes between virus strains. With the availability of deep sequencing technology, researchers have more recently begun to go deeper by comparing the viral quasispecies that are present in individual hosts. For example, the degree of intrahost variation of virus populations during SARS-CoV-2 and influenza infections, and the effect of bottlenecks associated with transmission between hosts, have recently been examined [25–28]. Interestingly, the findings with these other viruses are consistent with what we have observed, namely the presence of limited viral genome variation during acute infections in individual hosts, with strong negative selection and severe bottlenecks acting to limit the amount of diversity in these viruses as they spread through susceptible populations.

In this study, we explored whether there were differences between the virus populations in individual infected mosquitoes that could explain the tremendous amount of virus titer variation observed amongst highly inbred *Ae*. *aegypti* mosquitoes after oral infection with SINV. Our results revealed that there are both genetic differences at the population level and differences in the ratio of infectious units to genomes, or SI, between the virus populations found in low titer versus high titer mosquitoes. We did not find any evidence of specific candidate mutations that are associated with virus titer; instead, we found that the virus populations of low titer mosquitoes experienced significant reductions in genetic complexity and diversity during virus dissemination from the midgut, while those of high titer mosquitoes did not. There was also evidence of stronger purifying selection in both midguts and carcasses of low titer versus high titer mosquitoes. In addition, we observed dramatic differences in SI between low titer and high titer mosquitoes. Overall, our data indicate that there are both differences in the genetic composition and profound differences in SI between the virus populations found in low and high titer mosquitoes. At this point it is not clear whether these two types of observed differences are causally related, but based on our results, the huge variation in SINV titer between individual *Ae*. *aegypti* mosquitoes is best explained by differences in SI, likely due to a component of the immune response that directly or indirectly affects packaging of viral genomes into virions. Related to this, our results also provide important new insight into the mechanisms underlying previous demonstrations that antibiotic treatment suppresses mosquito antiviral immunity [22–24]. It should be noted that despite their being extensively inbred over the past ~80 years, the amount of genetic heterogeneity in Orlando mosquitoes is unknown, and it remains possible that there is genetic variation amongst individuals that affects their immune response.

The midgut, which is the initial site of infection, poses significant physical barriers to arbovirus infection [9]. Genetic bottlenecks occur during the process of midgut infection as well as during escape of virus from the midgut to the hemocoel. The number of viruses that manage to pass through these bottlenecks is likely quite small [10,11]. Thus, each genetic bottleneck has the potential to lead to founder effects, which can influence the eventual genetic structure of the virus population within an infected mosquito [10,12,13]. Our sequencing of viral populations indicated that there are higher numbers of genetic variants in the midguts than in the carcasses of both low and high titer mosquitoes, consistent with a midgut escape bottleneck. However, there were roughly similar numbers of genetic variants in the midguts of low and high titer mosquitoes, although some of the individual low titer midguts contained higher numbers. We did not observe a difference in the estimated bottleneck sizes between low and high titer mosquitoes, indicating that similar numbers of viruses escaped from the midgut in these individuals. Despite this, we found that low titer mosquitoes experienced a significant decrease in the number of variants during virus dissemination, while high titer mosquitoes did not. Measures of genetic complexity and diversity were also reduced following virus dissemination in low titer mosquitoes, but not in high titer individuals. In addition, there was also evidence of stronger negative or purifying selection observed in low titer mosquitoes than in high titer mosquitoes. Finally, previously published data showed that virus obtained from high titer mosquitoes replicated better than virus from low titer mosquitoes in cell culture [16]. All of these results are consistent with the conclusion that the virus populations in the midguts of low titer mosquitoes are overall less fit than those in the midguts of high titer mosquitoes. A likely source of this difference is genetic founder effect, occurring due to the midgut infection bottleneck. Despite the fact that the mosquitoes were all exposed to the same population of genetic variants present in the stock virus, stochastic effects caused by the midgut infection bottleneck may have led to some mosquitoes being infected with variants that were less fit than those infecting others.

To investigate this further, we examined the SI ratio (infectious units per genome equivalent) in orally infected mosquitoes. The results revealed that the huge variation in SINV infectious titer between individual mosquitoes is mainly due to differences in SI. The SI of SINV has been reported to vary somewhat depending on the cell line being infected, but to be in the range of 10^−1^ to 10^−3^ when infecting either BHK or C6/36 cells [29]. Thus, the SI values we observed in low titer mosquitoes, ranging from 10^−3^ to 10^−6^, are exceptionally low compared to what has been previously reported from infection of cell lines.

The infectivity of SINV that is produced in vertebrate cells has been shown to be affected by encapsidation of host factors, resulting in two populations of SINV particles that differ in density as well as infectivity [30, 31]. However, SINV produced in mosquito cells was found to only produce the more infectious type of these particles [31]. Furthermore, we observed decreased numbers of viral particles in low titer mosquitoes, making differences in encapsidation of host factors an unlikely source of the variation in infectivity observed in this study. Instead, it appears that viral RNA is not encapsidated efficiently in low titer mosquitoes, for reasons that are currently unclear. There are numerous possible explanations for why a large proportion of viral genomes are not being packaged in low titer mosquitoes. For example, viral RNA could be sequestered in cells, preventing its encapsidation. Translation of viral RNA could be less efficient, resulting in lower amounts of viral proteins; indeed, our immunoblotting results indicated that there were lower levels of capsid protein on average in low titer mosquitoes, although the levels of capsid protein varied and did not entirely correlate with titer. Related to this, translation defects could be due to differences in RNA modifications such as capping or methylation, as in Wolbachia-colonized mosquitoes where it has been shown that decreased cytosine methylation in SINV RNA leads to reduced infectivity [32,33]. It is also possible that one or more viral proteins are being modified somehow to make them less stable or less able to function, for example by altering their localization in cells. Unfortunately, we were not able to detect the viral glycoproteins E1 and E2 in individual mosquitoes in our immunoblots, but it is possible these proteins are mislocalized, unstable, or not functioning properly. However, it is notable that we did not observe unenveloped nucleocapsids by TEM, indicating the low production of infectious particles is not likely to be simply due to lack of envelopment of nucleocapsids. A significant clue as to what is affecting genome encapsidation is our observation that suppressing antiviral immunity using antibiotic treatment caused an increase in SI. Of course, additional work will be necessary to understand the precise underlying mechanisms causing inefficient genome packaging in low titer mosquitoes.

We further examined whether there were differences in SI between the virus populations in midguts and carcasses, and if SI changed over time in infected mosquitoes. Our results indicated that the virus found in midgut at 5 days PBM had an overall lower SI than virus in carcass. However, there was no correlation between the midgut SI and carcass SI in individual mosquitoes, arguing against inefficient genome packaging being due to specific genetic differences in the viral populations. Furthermore, SI as well as median titer decreased over time, with SI being lower at 15 days PBM than at 5 days. These results further hint at the possibility that there may be a host response that can somehow affect the packaging efficiency of SINV genomic RNA, and that this response is more effective in some mosquitoes than in others, leading to extensive variation in the titer of infectious virus. In addition, this putative immune-related response appears to be more active in the midgut than in the carcass and appears to have a cumulative effect on virus infectivity over time, and/or is increasingly active as time goes on.

In conclusion, our findings indicate that the huge amount of variation in SINV infectious titer that is observed between individual infected *Ae*. *aegypti* mosquitoes within a single experiment is due to differences between the virus populations found in different individuals. These include genetic differences at the population level, which may be the result of founder effects that occur due to genetic bottlenecks associated with midgut infection and escape. In addition, we observed profound differences in the efficiency with which viral genomes were efficiently packaged into viral particles between mosquitoes, apparently due to some type of mosquito response that can affect the efficiency of packaging of SINV genomic RNA, such as by sequestering or modifying viral RNA, inhibiting translation of the viral structural proteins, or affecting their stability, localization, or function. Whether the genetic differences between virus populations and the differences in SI are causally related or only correlated remains to be determined. It will also be of interest to determine whether similar differences in virus populations and specific infectivity are observed in low and high titer infections of other vector mosquitoes and in *Ae*. *aegypti* infected with other arboviruses, where high individual variability in virus titer has also been observed. Finally, our work highlights the importance of measuring titers of infectious virus when performing experiments in arbovirus-infected mosquitoes, since viral genome copy number may not always accurately reflect the amount of infectious virus present.

## Materials and methods

### Cell lines

BHK-21 cells used were maintained in Dulbecco modified Eagle medium (DMEM, Gibco, Waltham MA) supplemented with 10% fetal bovine serum (FBS, Atlanta Biologicals, Minneapolis, MN) at 37°C with 5% CO_2_. C6/36 cells were propagated in Leibovitz’s L-15 medium (Gibco) plus 10% FBS at 27°C. Aag2 cells were maintained at 27°C in Schneider’s medium (Gibco) plus 10% FBS.

### Mosquito rearing

*Ae*. *aegypti* Orlando strain (maintained in our insectary since they were obtained in 2005 from James Becnel, Agricultural Research Service, U.S. Department of Agriculture, Gainesville, FL, USA) were reared at 27°C, 80% humidity on a 12 h light/12 h dark cycle. *Ae*. *aegypti* eggs were hatched in water containing brain heart infusion media (BHI) for 2 days to produce mosquito larvae. Mosquito larvae were separated into pans (each pan containing approximately 100 larvae) with a small amount of BHI and ground fish meal added. Adult mosquitoes emerged beginning at around 2 days after pupation. Adults were fed on raisins and water before and after blood feeding. All mosquito experiments were conducted in an arthropod containment level 2 insectary at Kansas State University.

### Preparation of virus stocks

The MRE-16 infectious clone p5′dsMRE16ic was obtained from Ken Olson (Colorado State University), and its construction was previously described [34,35]. The p5′dsMRE16ic plasmid was linearized with Ascl. Capped viral RNA was produced from the linearized plasmids using m^7^G (5′)ppp(5′)G Cap Analog (Ambion, Austin TX) and MEGAscribe SP6 Transcription Kit (Thermo Fisher, Waltham MA). A density of 5 × 10^5^ cells BHK21 cells were plated in each well of 6-well plates in serum-free opti-MEM media (Gibco) and allowed to attach for 2 hours. Six μL of Lipofectamine 3000 (Thermo Fisher), 100 μL opti-MEM, and 10 μL of 13.5 ng/ul viral RNA were mixed and allowed to sit for 5 minutes. After incubation, 900 μL of opti-MEM was then added to the tube containing the mixture and mixed with the seeded BHK-21 cells [36]. After incubation at 37°C for 3 days, the medium was collected, aliquoted, and stored as P1 virus stock at −80°C. The P1 virus stocks were passaged by adding 200 μL of P1 virus to a T75 flask containing 90% confluent C6/36 cells and cultured in Leibovitz’s L-15 medium supplemented with 10% (vol/vol) FBS to obtain P2 viral stocks. At 5 days post-infection (dpi), the P2 virus was harvested, aliquoted, and stored at −80°C. Viral titers were determined using median tissue culture infectious dose (TCID_50_) assay in BHK-21 cells, as described below. Virus stocks were thawed only once before use.

### Oral infection with SINV

Newly emerged adult female mosquitoes were fed on raisins and water ad libitum. At 2 days post-emergence, raisins were removed but not water, and mosquitoes were starved for 1 day. The mosquitoes were then separated into smaller containers. P2 virus stock was mixed with defibrinated sheep blood (Colorado Serum Company, Denver CO) in a ratio of 1:1 and mosquitoes were allowed to feed for 1 hour using a Hemotek membrane feeder (Hemotek Ltd., Blackburn, UK) covered with Parafilm. The mosquitoes were briefly cold-shocked at 4°C, and then fully engorged females were separated from the remaining mosquitoes. Fed females were subsequently maintained on raisins and water until 5 days post-blood meal (PBM) before dissecting. Mosquitoes were dissected in phosphate-buffered saline (8 g NaCl, 0.2 g KCl, 1.44 g Na_2_HPO_4_, 0.24 g KH_2_PO_4_, 1 liter H_2_O) and midguts were removed from the carcasses. Mosquito midguts and carcasses were placed in 200 μL DMEM plus 10% FBS and homogenized in 1.5-mL microcentrifuge tubes using disposable plastic pestles. In cases where RNA extraction was performed, a volume of 180 μL was placed into 500 μL Trizol (ThermoFisher Scientific) solution. Both samples were frozen at −80°C until titration and RNA extraction.

### TCID_50_ Assay

A total of 1 × 10^4^ BHK-21 cells were seeded in each well of 96-well tissue culture plates. The DMEM media used in seeding the cells contained 10% FBS and was supplemented with Antibiotic-Antimycotic Solution (Caisson Labs). Frozen mosquito samples were thawed on ice and centrifuged at 4°C for 3 minutes at 15,000× g to remove debris. Serial dilutions of each sample were added to 5 duplicate wells of BHK-21 cells. The plates were scored for cytopathic effects after 5 days, and the proportion of infected wells was used to calculate TCID_50_/mL values [37].

### TCID_50_ assay in mosquito cells using immunofluorescence

Aag2 and C6/36 cells were plated in 96 well plates and incubated with 10-fold serial dilutions of virus stocks obtained from low or high titer mosquitoes (as previously determined using BHK cells). At 4 days post-infection, media was removed, 4% paraformaldehyde (ThermoFisher) was added to fix the cells and the plates were rocked for at least 2 hours at room temperature. The cells were then washed two times with 100 μL PBT (PBS + 0.05% Triton X-100). A volume of 50 μL anti-Sindbis E1 monoclonal antibody (mAb 30.11A, obtained from Stephen Higgs, Kansas State University) diluted 1:300 in PBT solution was added and rocked for 90 minutes at 25°C. The cells were then washed twice with PBT. A volume of 50 μL of anti-mouse IgG conjugated to Alexa488 (Life Technologies) diluted 1:300 in PBT was added and incubated for 60 minutes at 25°C with rocking, after which the wells were washed two times with PBT. The presence or absence of antibody staining in each well was observed using a fluorescence microscope, from which TCID_50_ values were determined.

### SINV genome quantification by RT-qPCR

Total RNA from mosquito midguts and carcasses was extracted using the PureLink RNA Mini Kit (Thermo Fisher Scientific) according to manufacturer instructions. Power SYBR Green RNA-C_T_ 1-Step Kit (Thermo Fisher Scientific) was used for the RT-PCR reaction according to the manufacturer instructions. The primers targeted the NSP3 region with sequences as follows: Forward primer 5’-AGAAGAGGCTTCAGGGCTGG-3’ and Reverse primer 5’-TGACGAGGTCTTTGGCTGTG-3’. The cycling conditions for the reaction were reverse transcription step at 48°C for 30 minutes, enzyme activation step at 96°C for 10 minutes, followed by 40 cycles of denaturation at 96°C for 15 seconds and annealing/extension at 56°C for 60 seconds. A standard curve was generated using a 1:10 dilutions of the p5’dsMRE16ic plasmid. Cycling threshold (Ct) values were used to determine number of RNA copies in each sample. The RT-qPCR experiment for each sample was carried out in duplicate and the results were averaged.

### Illumina sequencing

Six mosquito midgut and carcass samples each were selected for sequencing from low and high titer mosquitoes. RNA was extracted from the individual samples using PureLink RNA Mini Kit. The stranded mRNA-Seq was performed using the Illumina NovaSeq 6000 Sequencing System at the University of Kansas Medical Center–Genomics Core (Kansas City, KS). Quality control of the total RNA submissions was completed using the Agilent TapeStation 4200 using the RNA ScreenTape Assay kit (Agilent Technologies 5067–5576). Total RNA (228.06ng– 264.6ng) was used to initiate the library preparation protocol. The total RNA fraction was processed by oligo dT bead capture of mRNA, fragmentation of enriched mRNA, reverse transcription into cDNA, end repair of cDNA, ligation with the appropriate Unique Dual Index (UDI) adaptors and strand selection and library amplification by PCR using the Universal Plus mRNA-seq with NuQuant library preparation kit (Tecan Genomics 0520-A01).

Library validation was performed using the D1000 ScreenTape Assay kit (Agilent Technologies 5067–5582) on the Agilent TapeStation 4200. Concentration of each library was determined with the NuQuant module of the library prep kit using a Qubit 4 Fluorometer (Thermo Fisher), Libraries were normalized to 4nM concentration and pooled. The multiplexed pool was quantitated, in triplicate, using the Roche Lightcycler96 with FastStart Essential DNA Green Master (Roche 06402712001) and KAPA Library Quant (Illumina) DNA Standards 1–6 (KAPA Biosystems KK4903). Using the qPCR results, the RNA-Seq library pool was adjusted to 1.9nM for multiplexed sequencing.

Pooled libraries were denatured with 0.2N NaOH (0.04N final concentration) and neutralized with 400mM Tris-HCl pH 8.0. A dilution of the pooled libraries to 380 pM was performed in the sample tube, on instrument, followed by onboard clonal clustering of the patterned flow cell using the NovaSeq 6000 S1 Reagent Kit v1.5 (200 cycle) (Illumina 20028318). A 2x101 cycle sequencing profile with dual index reads was completed using the following sequence profile: Read 1–101 cycles x Index Read 1–8 cycles x Index Read 2–8 cycles x Read 2–101 cycles. Following collection, sequence data was converted from bcl file format to fastq file format using bcl2fastq software and de-multiplexed into individual sequences for data distribution using a secure FTP site or Illumina BaseSpace for further downstream analysis.

### Illumina sequence data analysis

NGS data was processed and analyzed essentially as described elsewhere [38]. Briefly, trimmed reads from fastq files were aligned to the reference 5’dsMRE16ic genome and duplicates removed prior to calling intrahost single nucleotide variants (iSNVs) using Vphaser2 [39]. iSNVs with significant strand bias or those present in the population at less than five percent frequency were removed. Sequence data have been deposited in the Sequence Read Archive under BioProject ID PRJNA1000578.

### Bottleneck size estimation

Estimation of the bottleneck size at the initial midgut site of infection was calculated by analyzing the change in frequency distribution of neutral markers between stock virus in bloodmeal (initial) and midgut infection (final) sample. The single nucleotide variants (SNVs) that were presumed to be neutral were selected based on the following set of criteria: (i) change should occur at the third codon position, (ii) this change should be synonymous, and (iii) the SNVs should be found in virus in blood meal, low titer midgut and high titer midgut samples [40]. Assuming neutrality (i.e., in the absence natural selection), the idealized number of founding genomes (N) initiating midgut infection was determined using the formula

$$N=\frac{p(1-p)}{Var(p^{\prime })-Var(p)}$$

where p is the marker allele frequency of the stock virus in blood (initial population) and p′ is the marker allele frequency of the infected midgut virus population (final population) [40].

### Calculation of mutation frequency

The mutation frequency was calculated for each ORF as the number of substitutions per nucleotide, averaged for the 6 mosquitoes of each sample type.

### Natural selection assessment

The natural selection was assessed using the *d*_*N*_*/d*_*S*_ ratio. The number of non-synonymous substitutions per non-synonymous site (*d*_*N*_) and number of synonymous substitutions per synonymous site (*d*_*S*_) were calculated using:

$$d_{N}=\frac{-3\;\ln\left(1-(4p_{n})/3\right)}{4}$$

and

$$d_{S}=\frac{-3\;\ln\left(1-(4p_{s})/3\right)}{4}$$

where *p*_*n*_ is the number of non-synonymous substitutions divided by the number of non-synonymous sites and *p*_*s*_ is the number of synonymous substitutions divided by the number of synonymous sites [41]. DnaSP software was used to calculate the synonymous and non-synonymous sites.

### Virus complexity and diversity analysis

SINV population genetic complexity was estimated using normalized Shannon entropy (Sn) for each nucleotide site using the formula [42]:

$$Sn=-\frac{pln(p)+(1-p)\ln\left(1-p\right)}{\ln\left(4\right)}$$

where p is the minor allele frequency at the considered position, and ln(4) corresponds to maximum complexity (i.e., four possible nucleotides at each position). For individual populations, Sn values range from 0 to 1 where 0 represents no complexity while 1 represents maximum complexity.

SINV population genetic diversity was estimated using nucleotide diversity at each nucleotide site using the formula [43]:

$$\pi =(\frac{D}{D-1})(1-(p^{2}+{\left(p-1\right)}^{2})$$

where D is the sequencing depth at the considered position and p is the SNV minor allele frequency. For individual SNVs, π values range from 0 to 1 where 0 represents no diversity while 1 represents maximum diversity.

### Transmission electron microscopy

Low titer (3.2x10^3^–4.0x10^4^ TCID_50_/ml), medium titer (1.5x10^5^–3.0x10^6^ TCID_50_/ml, and high titer (1.9–4.5x10^8^ TCID_50_/ml) mosquitoes (3 of each) were individually homogenized in PBS and insoluble debris was removed by centrifugation in a microcentrifuge. The homogenates were fixed with 2% paraformaldehyde at room temperature for 2 hrs, negative stained with uranyl acetate, absorbed onto grids, and viewed by transmission electron microscopy. The entirety of each grid was scanned at low magnification to identify possible virions, followed up by examination at higher magnification to verify the presence of virions that were similar in size and shape to SINV.

### Immunoblotting

Individual mosquitoes were homogenized in 200 μL of PBS and centrifuged in a microcentrifuge at 12,000 RPM for 5 min to remove insoluble material. Following removal of 20 μL of the lysate for TCID_50_ assay, 80 μL Laemmli SDS-Sample Buffer, 6X Reducing (Boston Bioproducts) was added. The samples were then stored at 4°C until being analyzed. Samples were heated for 5 min at 95°C, and then loaded on a 12% Tris-Glycine gel (BioRad). Gels were transferred to nitrocellulose membrane using an iBlot2 (ThermoScientific) and blocked using non-protein blocking buffer (Li-Cor). Blots were incubated while rocking overnight at 4°C in primary antibody (1:3000 anti-capsid, 1:3000 anti-glycoprotein, 1:1000 β-actin (SantaCruz)) and then washed and probed with IRDye800RD anti-rabbit for capsid and glycoprotein, and IRDye680RD anti-mouse for β-actin. Preparation of anti-capsid and anti-glycoprotein antisera was described previously [44]. Images were obtained using a ChemiDoc system (Bio-Rad Laboratories) and bands were quantified using Gel Analyzer in the FIJI software package [45] with background correction.

### Saliva collection and titration

Saliva was collected from 60 mosquitoes at 5 days PBM. Mosquitoes were starved of a sugar source for 24 h before saliva collection, then anesthetized by cold treatment, and their wings and legs were removed. The proboscis was placed in a pipette tip containing ∼20μL FBS + 1 mM ATP. Mosquitoes were allowed to salivate for 60–90 min. After salivation the FBS was added to 100μL DMEM. Samples were vortexed, spun down, and then stored at -80°C. Meanwhile, the mosquito bodies were assayed by TCID_50_, and the matching saliva samples from 13 high titer (≥1 x 10^8^ TCID_50_/ml) and 12 low titer (≤ 1 x 10^4^ TCID_50_/ml) mosquitoes were then assayed by TCID_50_.

### Antibiotic treatment

Pupae were randomly divided into 2 groups. The antibiotic treatment group was put in a pan with deionized H_2_O containing an antibiotic cocktail consisting of 100 μg/mL each of penicillin and streptomycin, and 100 units/mL of gentamycin (Gibco). The control group was transferred to a separate pan without antibiotics. After eclosion, adults in the antibiotic treatment group were given access *ad libitum* to a 10% sucrose solution containing the same antibiotic cocktail as above, while control adults were maintained on 10% sucrose without antibiotics. At 2 days post-eclosion, the sucrose solutions were removed from both treatments and at 3 days post-eclosion, the antibiotic group was allowed to feed on a mixture of blood and virus containing the same antibiotic cocktail while the control group was allowed to feed on blood and virus alone. The mosquitoes were dissected at 5 days PBM and carcasses were stored in 200μl of DMEM at -80°C until being assayed.

### Statistical analysis

GraphPad Prism 5 software was used for statistical analysis. Fisher’s exact, Mann–Whitney or Pearson’s chi-square tests were used to determine statistically significant differences between treatments. A p-value of ≤ 0.05 was used as the threshold to indicate significant difference between treatments.

## Supporting information

S1 Fig Genome coverage of the SINV genome in samples sequenced in the study. Each sequencing reaction was loaded in two lanes, resulting in two data points per sample. On average, carcass genome coverage was higher than midgut coverage. Blood fed (BF) controls were midguts and carcasses obtained from uninfected negative control mosquitoes that were given a blood meal lacking SINV.
(PDF)

S2 Fig Numbers of (A) deletion and (B) insertion variants present at ≥5% frequency in midguts and carcasses of low and high titer mosquitoes.
(PDF)

S3 Fig Sequence variants present at ≥5% frequency in P2 stock virus. (PDF)

S4 Fig Venn diagrams showing number of unique and shared open reading frame mutations between low titer mosquitoes and high titer mosquitoes. LTM, low titer midgut; HTM, high titer midgut; LTC, low titer carcass; HTC, high titer carcass.
(PDF)

S5 Fig Synonymous and non-synonymous nucleotide substitutions present in the ORF sequences of the virus populations from (A) midguts and (B) carcasses of low and high titer mosquitoes, shown as the proportion of total nucleotide substitutions in the viral genome.
(PDF)

S6 Fig Variants present at ≥5% frequency that were (A) conserved and (B) emerged variants in low titer and high titer mosquitoes. Conserved variants were those present in both midgut and carcass, while emerged variants were present in the carcass but absent in the midgut.
(PDF)

S7 Fig Midgut bottleneck size estimation using nucleotide positions (A) 149, (B) 4088, and (C) 5639 in the viral genome.
(PDF)

S8 Fig Titration of infectious virus using mosquito C6/36 and Aag2 cells. Low titer and high titer stocks (6 of each) that had previously been titered by TCID_50_ in BHK cells were re-titered using an immunofluorescence-based TCID_50_ assay in C6/36 and Aag2 cells.
(PDF)

S9 Fig Example images of SINV-like virus particles in high titer mosquito homogenates obtained by negative staining and TEM. (A) Two images that were obtained at different magnification (note scale bars below). (B) Estimated sizes are shown of the particles in (A), as measured using Image J software.
(PDF)

S10 Fig Immunoblots of lysates prepared from 7 high (≥1.8x10^8^ TCID_50_/mL) and 7 low (≤6.8x10^4^ TCID_50_/mL) titer mosquitoes probed with anti-capsid antibody or anti-actin antibody as a loading control. The migration of size markers is indicated on the left. Arrows indicate migration of the proteins of interest. (+) cntl, positive control SINV stock; Ladder, mw size markers (in KDa); BF, blood-fed uninfected control mosquitoes. Individual mosquito samples are indicated by their number labels starting with P. The blots shown are representative of consistent results obtained by blotting the same lysates 4 times. Shown below are the results of quantification of the capsid bands in each sample after normalization to actin.
(PDF)

S11 Fig Virus load in mosquito midguts versus carcasses (data corresponding to Fig 8). (A) Overall virus titers and genome equivalents in midguts and carcasses (n = 50). (B) Virus load in midguts, arranged in ascending order of midgut titer. (C) Virus load in carcasses, arranged in ascending order of carcass titer.
(PDF)

S12 Fig Specific infectivity of mosquitoes at 5 days compared to 15 days PBM. Mann-Whitney test was used for statistical analysis. n = 50 for 5 days and n = 30 for 15 days. The data used for 5 days PBM was obtained from the experiment shown in Fig 7.
(PDF)

S1 Table Genomic boundaries positions of ORFs and other genome elements in the 5’dsMRE16ic genome. (PDF)

S2 Table Numbers of virus particles similar in size and shape to SINV observed in homogenates from high, medium and low titer mosquitoes. Multiple fields of view were scanned for each sample, covering the entire TEM grid. Titer ranges were as follows: high titer, 1.9 x10^8^–4.5x10^8^ TCID_50_/mL; medium titer 1.5 x10^5^–3.0x x10^6^ TCID_50_/mL; low titer 3.2x10^3^-4.0x10^4^ TCID_50_/mL.
(PDF)

S1 Data This file contains the nucleotide variants detected at ≥5% frequency within the ORFs and the non-coding regions of the low titer midgut, low titer carcass, high titer midgut, high titer carcass, and stock virus sequences. The data are arranged in tabs according to sample type and variant position (coding versus non-coding). For variants found in coding regions, the original amino acid coded by the affected codon, the change in amino acid caused by the variant (if any), the amino acid position within the ORF and the ORF identity, and whether it is a synonymous or non-synonymous change are also listed for each variant. For non-coding variants, the affected genome element is listed.
(XLSX)

S1 Raw Data This file contains the raw numerical data used to construct the graphs in Figs 1–9. (XLSX)

S2 Raw Data This file contains the raw numerical data used to construct the graphs in S1–S12 Figs (with the exception of S4 Fig, which already has the data displayed in the figure, and S9 Fig, which does not contain any numerical data). (XLSX)
