# Supplementary figures and images for: Extreme infectious titer variability in individual Aedes aegypti mosquitoes infected with Sindbis virus is associated with both differences in virus population structure and dramatic disparities in specific infectivity
Source: PLoS Pathog. 2024 Feb 27;20(2):e1012047. doi: 10.1371/journal.ppat.1012047 (PMC10923411; doi:10.1371/journal.ppat.1012047)

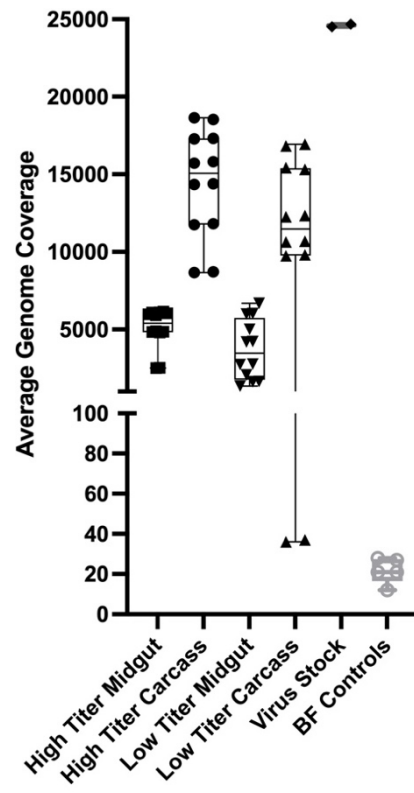

Supplement: S1 Fig — Each sequencing reaction was loaded in two lanes, resulting in two data points per sample. On average, carcass genome coverage was higher than midgut coverage. Blood fed (BF) controls were midguts and carcasses obtained from uninfected negative control mosquitoes that were given a blood meal lacking SINV. (PDF) [file ppat.1012047.s001.pdf]

**A**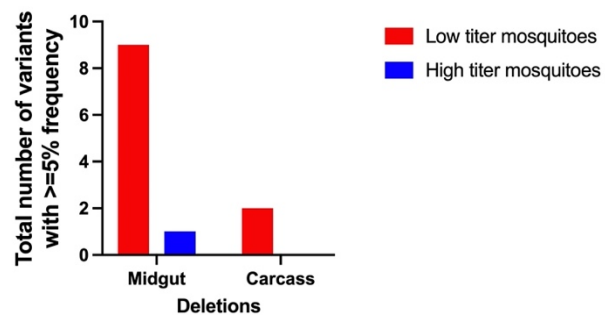**B**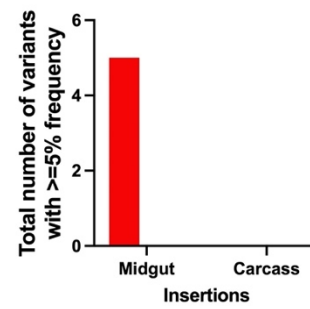

Supplement: S2 Fig — Numbers of (A) deletion and (B) insertion variants present at ≥5% frequency in midguts and carcasses of low and high titer mosquitoes. (PDF) [file ppat.1012047.s002.pdf]

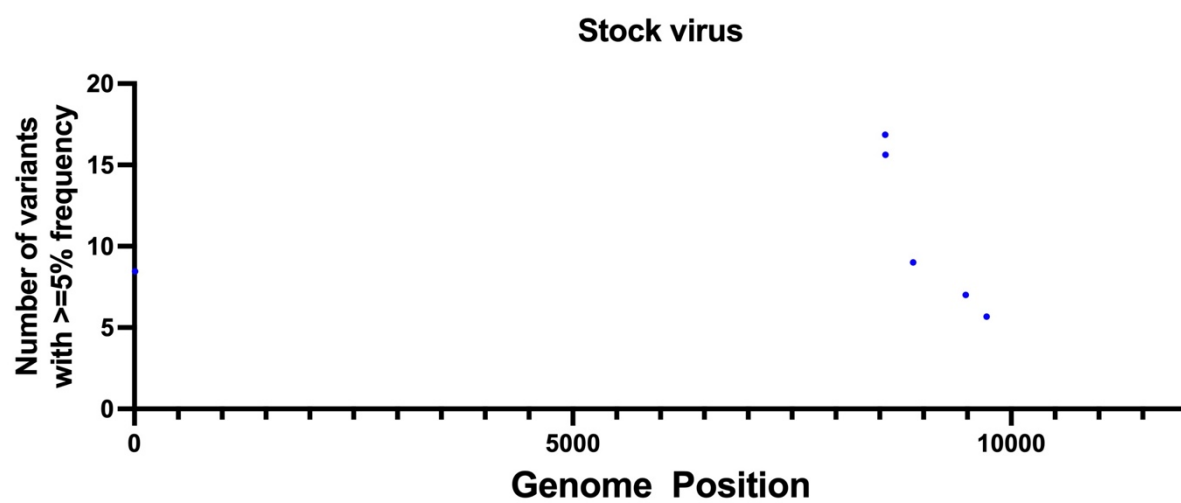

Supplement: S3 Fig — (PDF) [file ppat.1012047.s003.pdf]

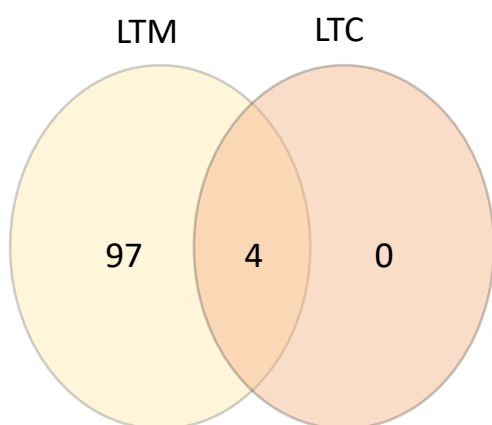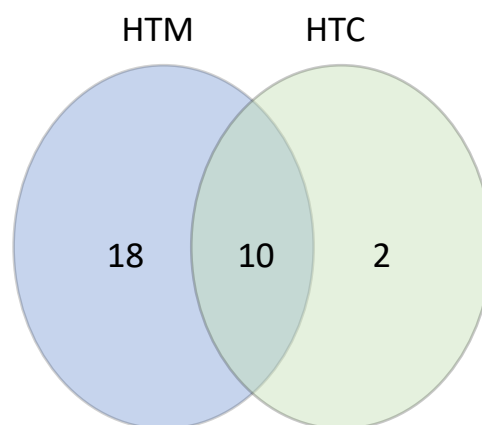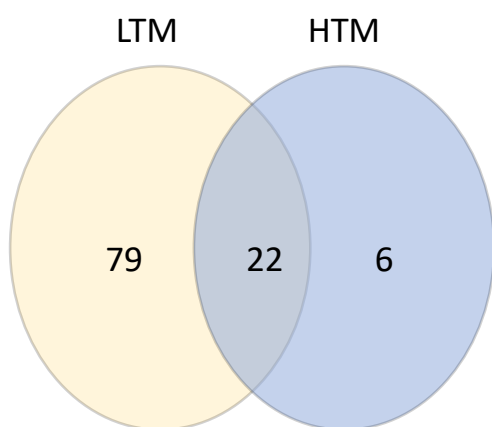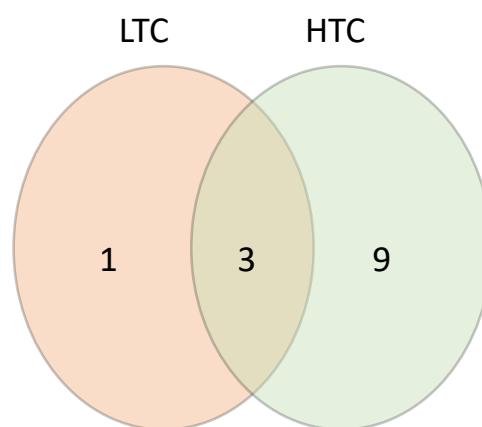

Supplement: S4 Fig — LTM, low titer midgut; HTM, high titer midgut; LTC, low titer carcass; HTC, high titer carcass. (PDF) [file ppat.1012047.s004.pdf]

**A**

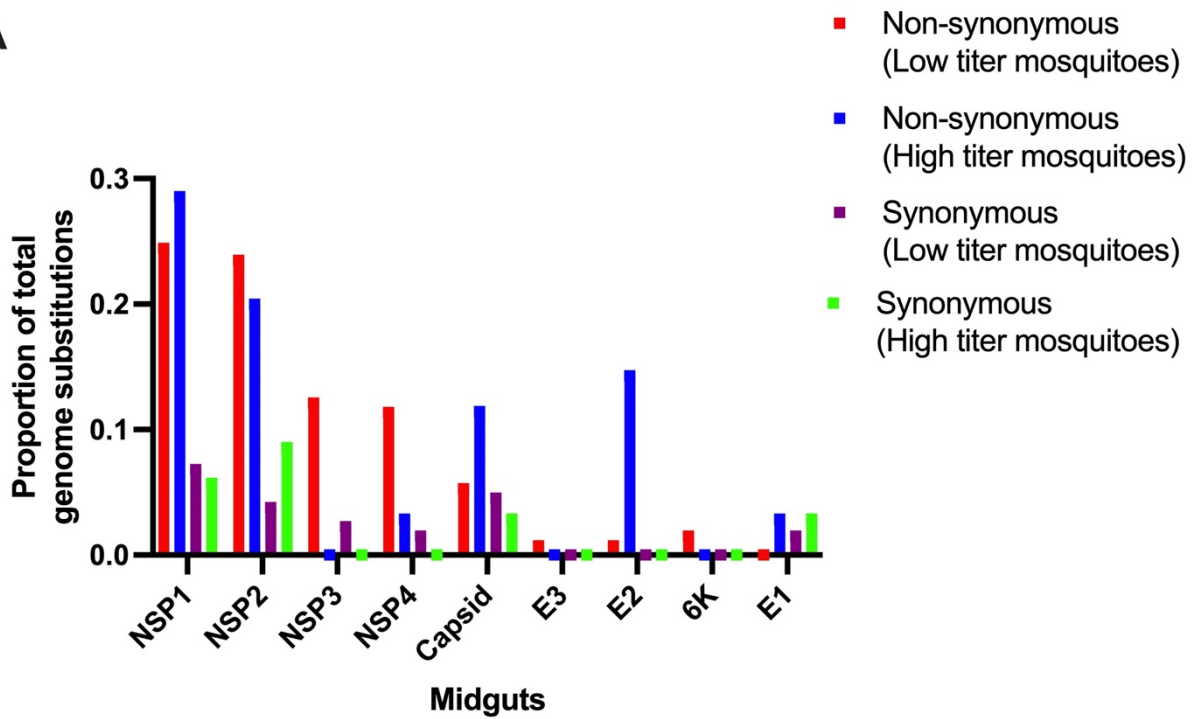

**B**

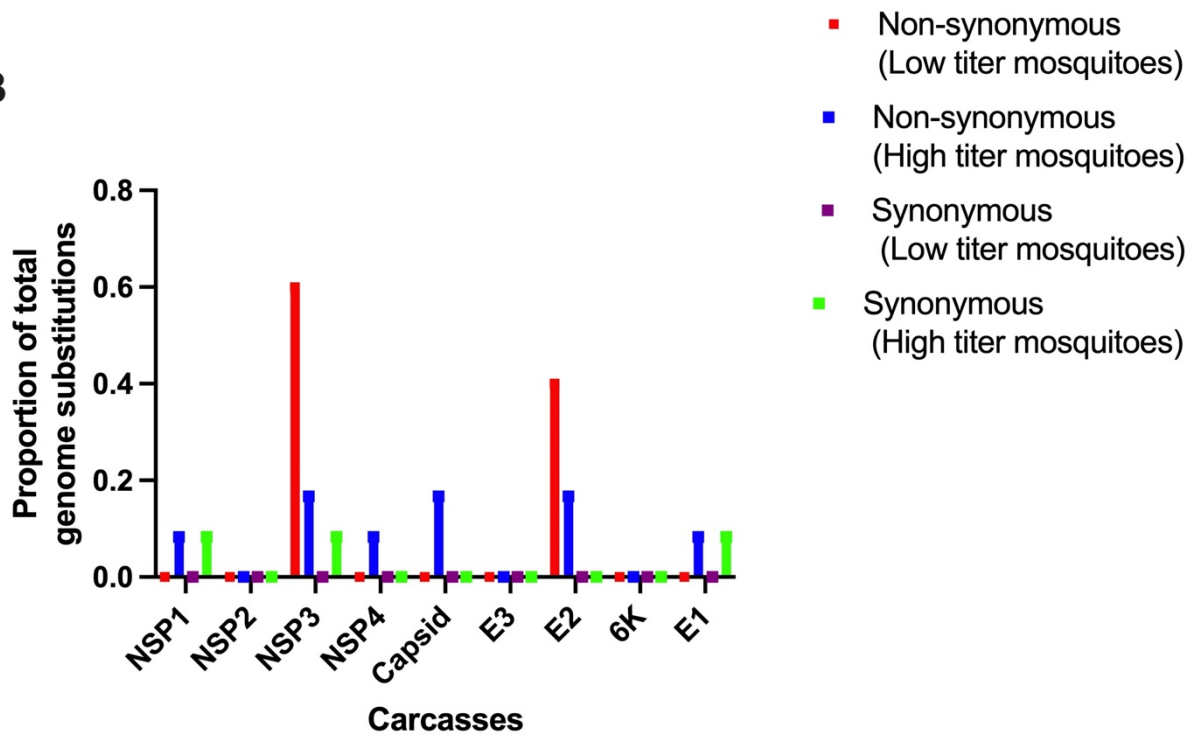

Supplement: S5 Fig — Synonymous and non-synonymous nucleotide substitutions present in the ORF sequences of the virus populations from (A) midguts and (B) carcasses of low and high titer mosquitoes, shown as the proportion of total nucleotide substitutions in the viral genome. (PDF) [file ppat.1012047.s005.pdf]

**A**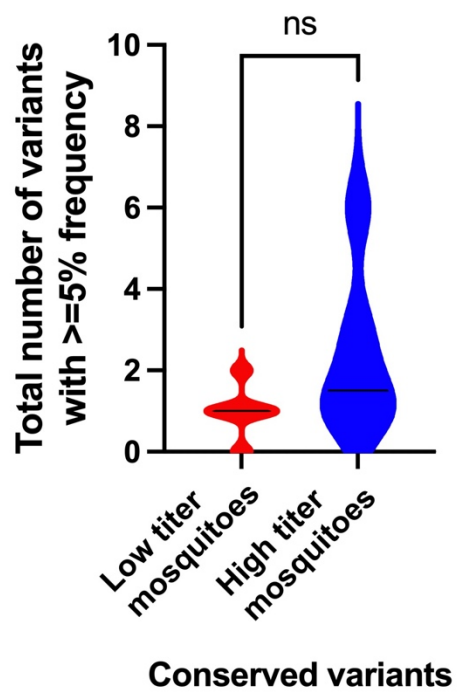**B**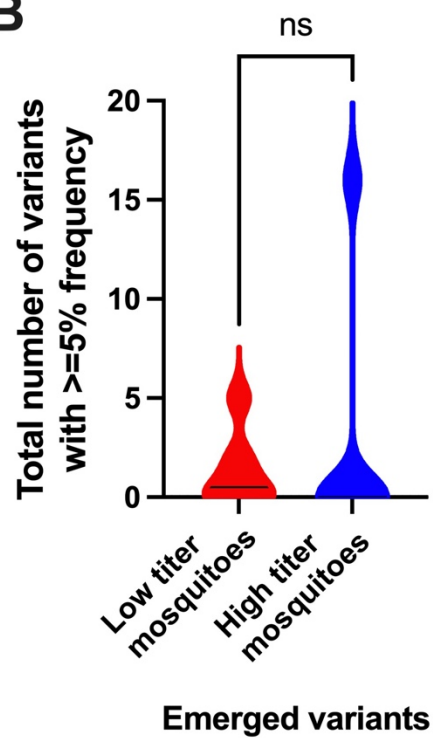

Supplement: S6 Fig — Variants present at ≥5% frequency that were (A) conserved and (B) emerged variants in low titer and high titer mosquitoes. Conserved variants were those present in both midgut and carcass, while emerged variants were present in the carcass but absent in the midgut. (PDF) [file ppat.1012047.s006.pdf]

**A**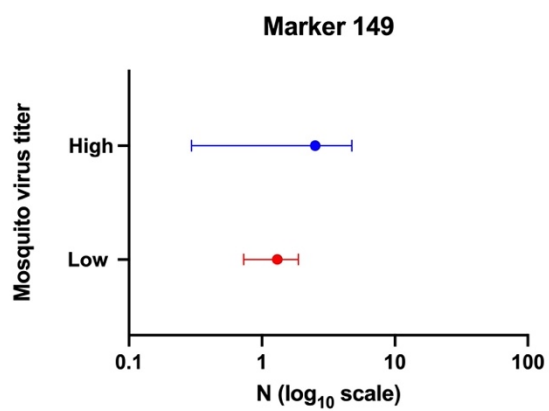**B**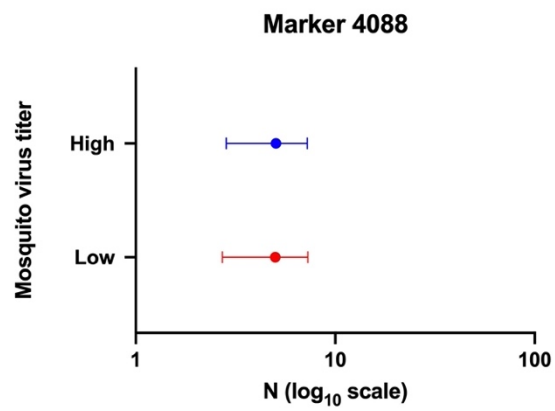**C**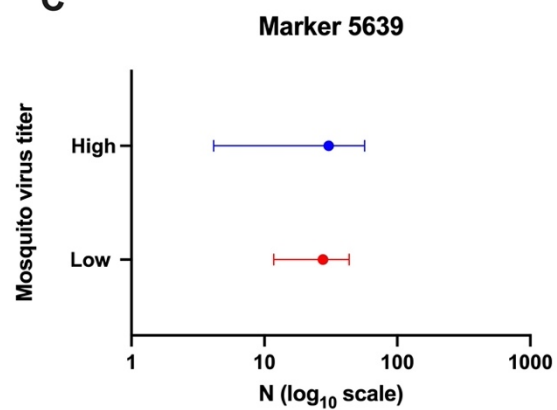

Supplement: S7 Fig — Midgut bottleneck size estimation using nucleotide positions (A) 149, (B) 4088, and (C) 5639 in the viral genome. (PDF) [file ppat.1012047.s007.pdf]

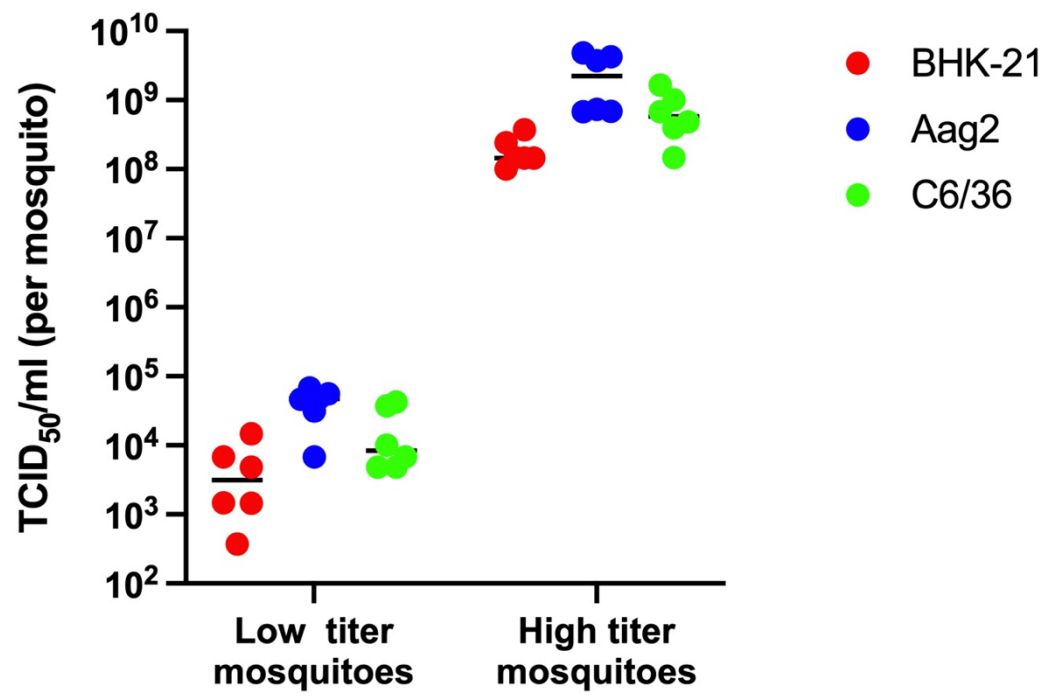

Supplement: S8 Fig — Low titer and high titer stocks (6 of each) that had previously been titered by TCID50 in BHK cells were re-titered using an immunofluorescence-based TCID50 assay in C6/36 and Aag2 cells. (PDF) [file ppat.1012047.s008.pdf]

A

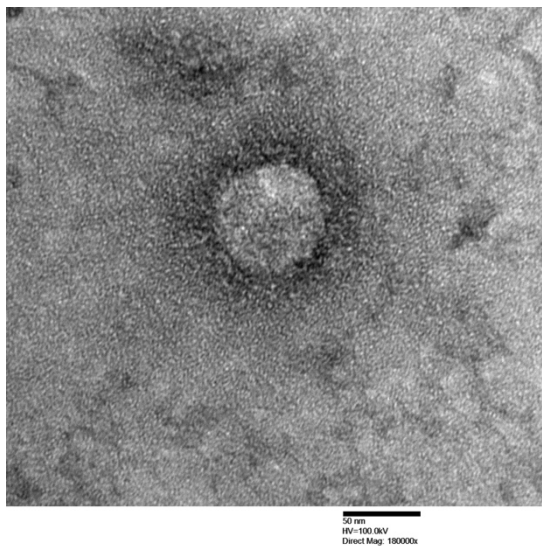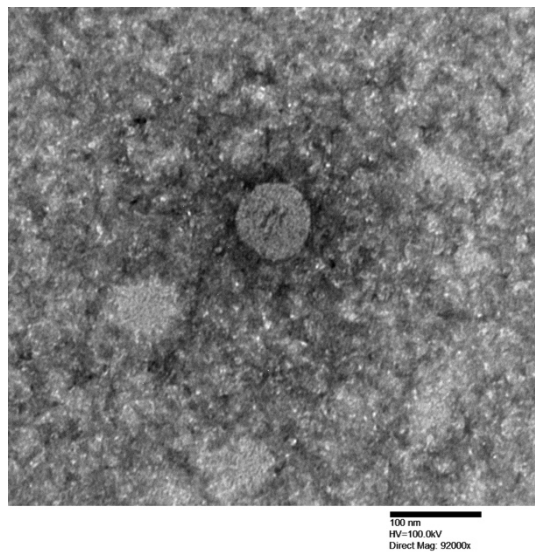

B

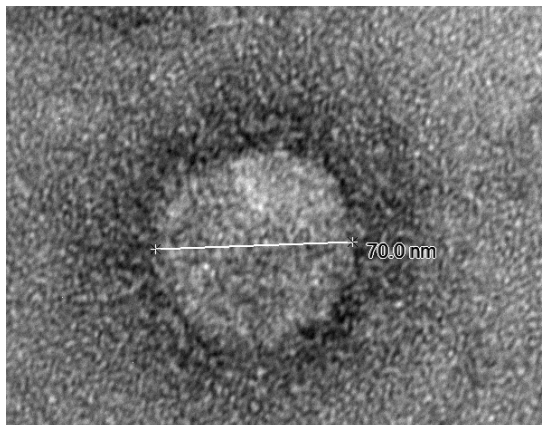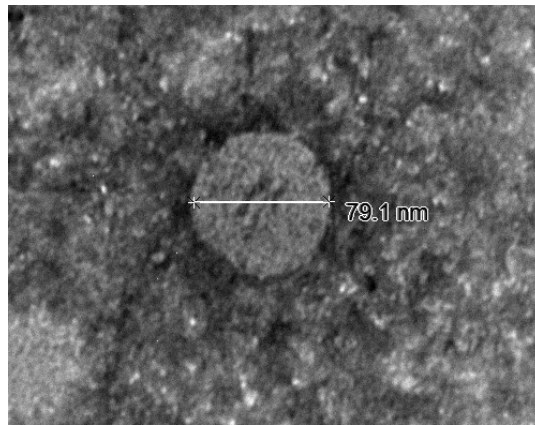

Supplement: S9 Fig — (A) Two images that were obtained at different magnification (note scale bars below). (B) Estimated sizes are shown of the particles in (A), as measured using Image J software. (PDF) [file ppat.1012047.s009.pdf]

## HIGH

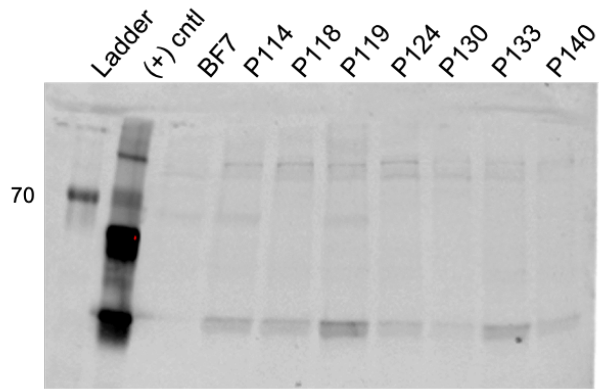

## LOW

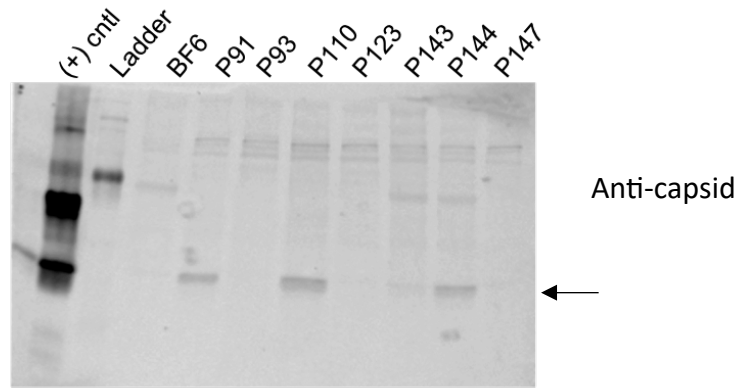

Anti-capsid

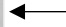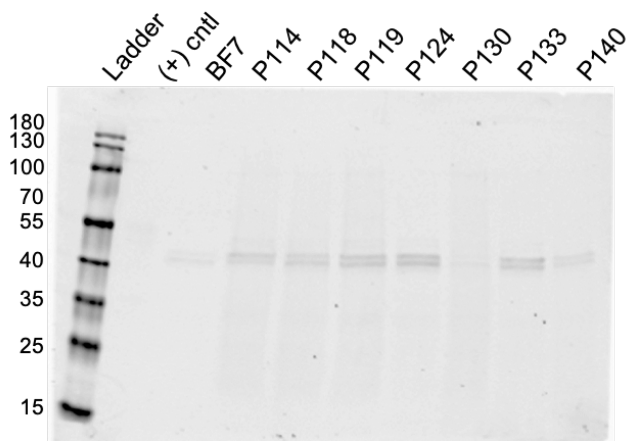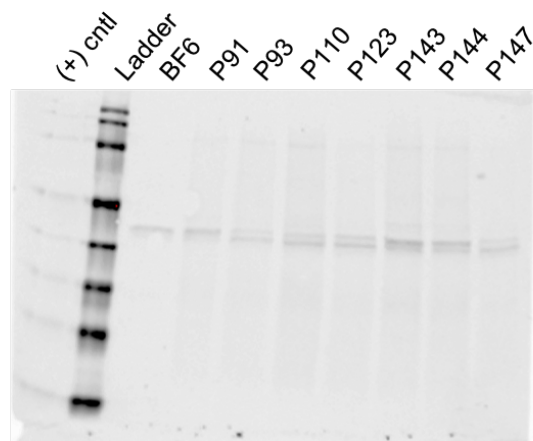

Anti-actin

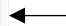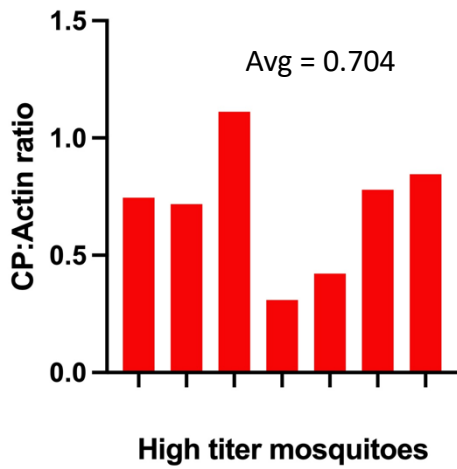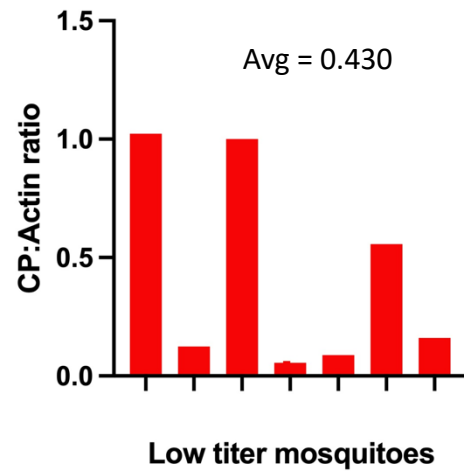

Supplement: S10 Fig — The migration of size markers is indicated on the left. Arrows indicate migration of the proteins of interest. (+) cntl, positive control SINV stock; Ladder, mw size markers (in KDa); BF, blood-fed uninfected control mosquitoes. Individual mosquito samples are indicated by their number labels starting with P. The blots shown are representative of consistent results obtained by blotting the same lysates 4 times. Shown below are the results of quantification of the capsid bands in each sample after normalization to actin. (PDF) [file ppat.1012047.s010.pdf]

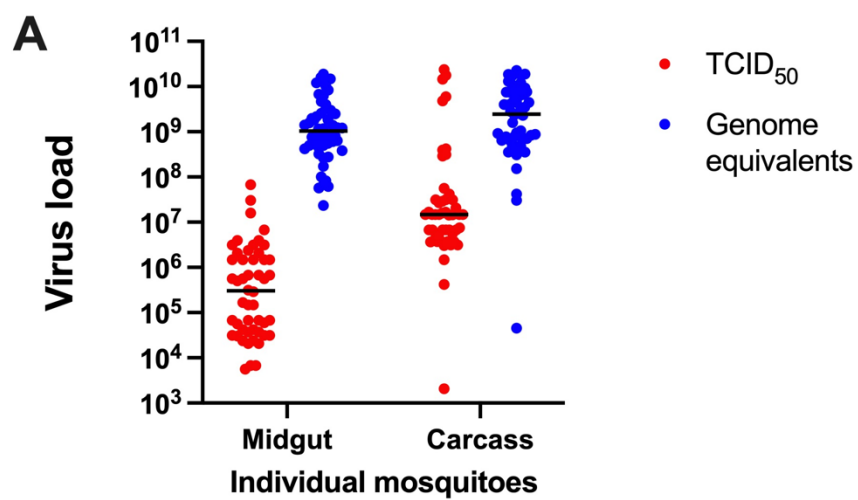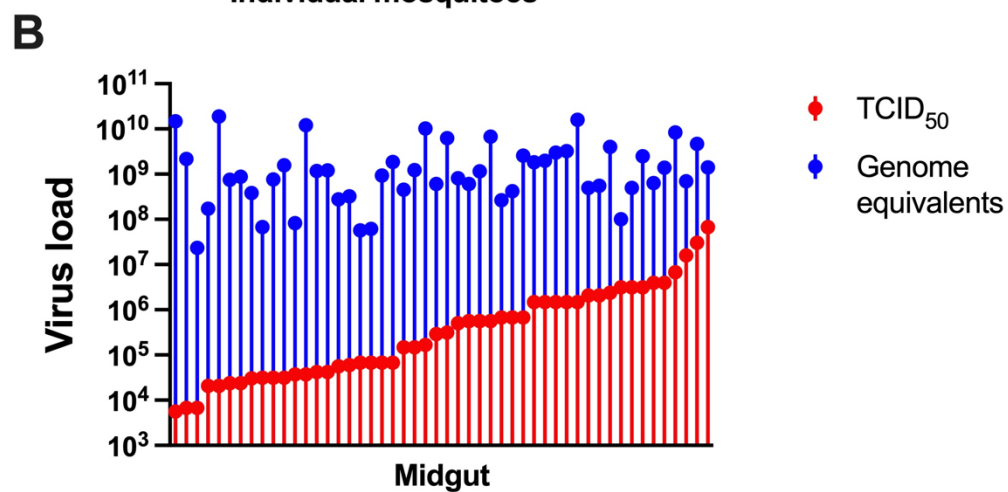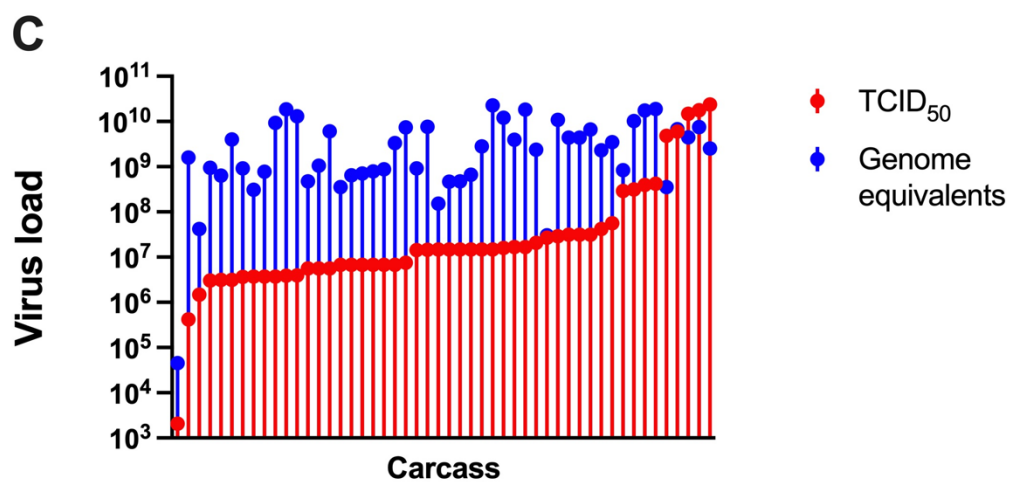

Supplement: S11 Fig — (A) Overall virus titers and genome equivalents in midguts and carcasses (n = 50). (B) Virus load in midguts, arranged in ascending order of midgut titer. (C) Virus load in carcasses, arranged in ascending order of carcass titer. (PDF) [file ppat.1012047.s011.pdf]

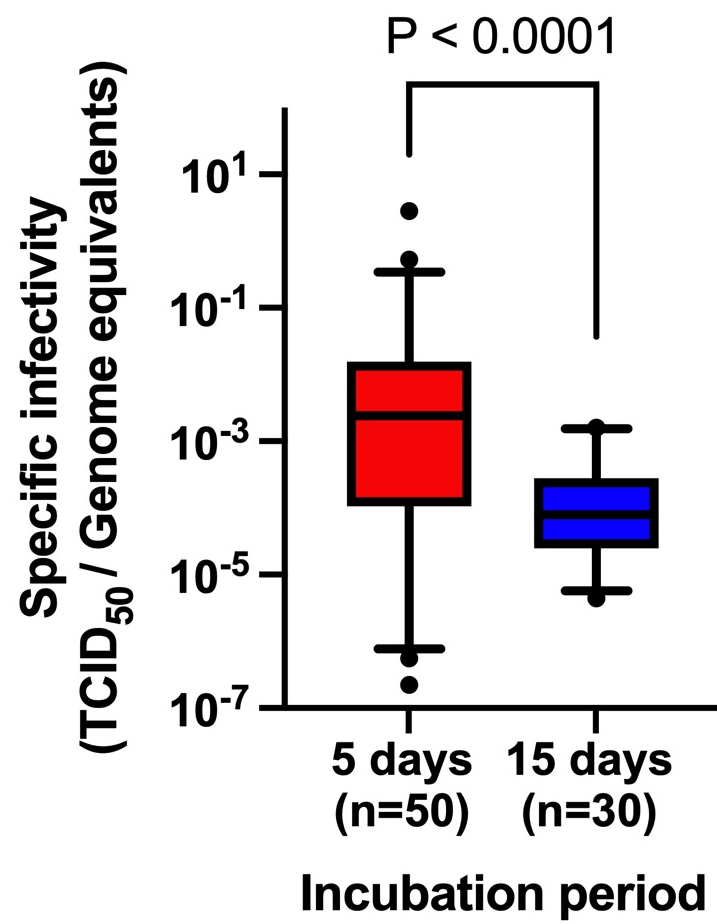

Supplement: S12 Fig — Mann-Whitney test was used for statistical analysis. n = 50 for 5 days and n = 30 for 15 days. The data used for 5 days PBM was obtained from the experiment shown in Fig 7. (PDF) [file ppat.1012047.s012.pdf]
