# Supplementary material for: Extreme infectious titer variability in individual Aedes aegypti mosquitoes infected with Sindbis virus is associated with both differences in virus population structure and dramatic disparities in specific infectivity
Source: PLoS Pathog. 2024 Feb 27;20(2):e1012047. doi: 10.1371/journal.ppat.1012047 (PMC10923411; doi:10.1371/journal.ppat.1012047)
Supplement: S1 Table — (PDF) [file ppat.1012047.s013.pdf]

S1 Table. Genomic boundary positions of ORFs and other genome elements in the 5'dsMRE16ic genome.

**Sequence**	**Start**	**End**
5'UTR	1	59
NSP1	60	1679
NSP2	1680	4100
NSP3	4101	5735
NSP4	5736	7484
subgenomic promoter region	7485	7795
Capsid	7796	8592
E3	8593	8784
E2	8785	10055
6K	10056	10219
E1	10220	11539
3'UTR	11540	11858
