# Supplementary material for: Extreme infectious titer variability in individual Aedes aegypti mosquitoes infected with Sindbis virus is associated with both differences in virus population structure and dramatic disparities in specific infectivity
Source: PLoS Pathog. 2024 Feb 27;20(2):e1012047. doi: 10.1371/journal.ppat.1012047 (PMC10923411; doi:10.1371/journal.ppat.1012047)
Supplement: S2 Table — Multiple fields of view were scanned for each sample, covering the entire TEM grid. Titer ranges were as follows: high titer, 1.9 x10^8^–4.5x10^8^ TCID50/mL; medium titer 1.5 x10^5^–3.0x x106 TCID50/mL; low titer 3.2x10^3^-4.0x10^4^ TCID50/mL. (PDF) [file ppat.1012047.s014.pdf]

S2 Table. Numbers of virus particles similar in size and shape to SINV observed in homogenates from high, medium and low titer mosquitoes. Multiple fields of view were scanned for each sample, covering the entire TEM grid. Titer ranges were as follows: high titer, 1.9 $x10^8$ - $4.5x10^8$ $TCID_{50}$/mL; medium titer 1.5 $x10^5$ -3.0x $x10^6$ $TCID_{50}$/mL; low titer $3.2x10^3$-$4.0x10^4$ $TCID_{50}$/mL.

SAMPLE TYPE AND ID	NUMBER OF PARTICLES OBSERVED
**HIGH TITER**	
**EL4**	>10
**EL7**	>10
**EL55**	>10
**MEDIUM TITER**	
**EL57**	0
**EL94**	0
**EL6**	0
**LOW TITER**	
**EL2**	1
**EL19**	1
**EL59**	0
